# Identification of a new population of Tnn^+^ progenitors to form tendon enthesis fibrocartilage

**DOI:** 10.1038/s41413-026-00519-3

**Published:** 2026-04-21

**Authors:** Tao Zhang, Lin Zhang, Ziyang Yuan, Linfeng Wang, Jianzhong Hu, Thomas Skutella, Hongbin Lu

**Affiliations:** 1 Department of Sports Medicine, Xiangya Hospital, Central South University, Changsha, China; 2 Key Laboratory of Organ Injury, Aging and Regenerative Medicine of Hunan Province, Changsha, China; 3 National Clinical Research Center for Geriatric Diseases, Xiangya Hospital, Changsha, China; 4 Department of Neuroanatomy, Group for Regeneration and Reprogramming, Institute for Anatomy and Cell Biology, Medical Faculty, Heidelberg University, Heidelberg, Germany; 5 Department of Spine Surgery and Orthopaedics, Xiangya Hospital, Central South University, Changsha, China

**Keywords:** Bone quality and biomechanics, Bone

## Abstract

Elucidating the identity of enthesis-resident progenitors is critical for advancing regenerative strategies, particularly in the context of the long-standing question of how is fibrocartilage formed at tendon enthesis (bone-tendon interface) under mechanical loading. To address the question of cellular origins of entheseal fibrocartilage, we first employed spatial transcriptional and single cell sequencing to identify a novel population of *Tnn*⁺ progenitor cells and delineate their lineage trajectories across developmental stages. Subsequently, we used a diphtheria toxin mediated ablation model targeting these *Tnn*⁺ progenitors and demonstrated their functional importance, as ablation resulted in hypoplastic phenotypes characterized by impaired fibrocartilage maturation. Furthermore, comparative single-cell profiling between unloaded entheses and normal entheses revealed that tendon unloading significantly diminished both the abundance and chondrogenic potential of *Tnn*⁺ progenitors. Collectively, these findings resolve fundamental questions regarding enthesis morphogenesis and provide mechanistic insights into how mechanical loading orchestrates this critical developmental process.

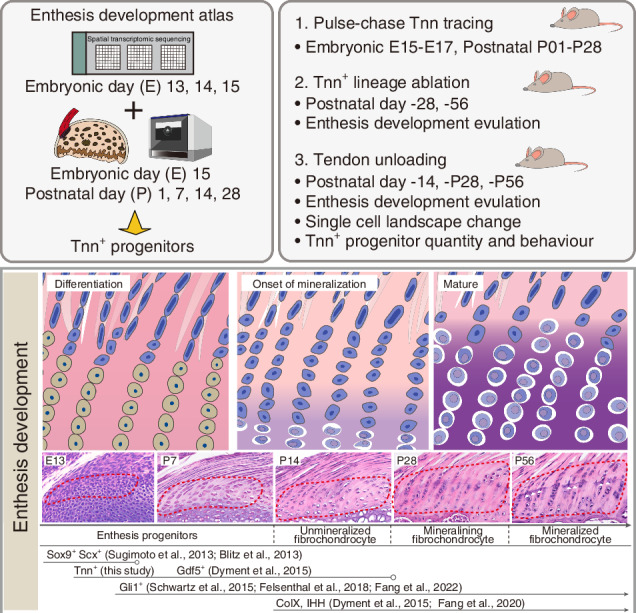

## Introduction

The tendon enthesis (bone-tendon interface) constitutes the specialized junction anchoring tendons to skeletal elements, serving dual mechanical roles in securing soft tissue attachments and transmitting musculoskeletal forces to osseous surfaces.^1,2^ Histologically, this transitional zone is characterized by a gradient of fibrocartilaginous layer exhibiting spatial gradients in cellular organization and mineralization patterns.^3^ Functionally, this graded architecture enables progressive modulus matching between dissimilar tissues - dissipating stress concentrations while maintaining structural continuity.^3,4^ Clinically, entheseal complexes (e.g., rotator cuff, Achilles tendon, patellar ligament) demonstrate particular vulnerability to traumatic rupture and post-surgical failure, with current regenerative approaches showing limited capacity to restore native fibrocartilaginous organization.^5^ This challenge stems from two fundamental questions: how is enthesis fibrocartilage formed, and what are the cellular origins that drive the formation of its mineral gradient?^6^ Answering these questions is essential for informing promising regenerative strategies.

The paradigm that enthesis regeneration recapitulates developmental processes has achieved scientific consensus, creating compelling rationale for characterization of enthesis-resident progenitor populations.^6^ While substantial advances have been made in understanding the progenitor biology across musculoskeletal tissues,^7–9^ the enthesis progenitors which dominate the growth of the fibrocartilage remain enigmatic. The inherent tissue complexity arising from tri-lineage integration (osteogenic, chondrogenic, and tenogenic) creates a cellular heterogeneity that obscures developmental mapping efforts.^6^ Thankfully, recent studies employing single cell RNA sequencing (scRNA-seq) have shed light on the enthesis developing mysteries.^10–12^ In late embryonic stage, the tendon enthesis is initially united as bi-fated to express both the chondrogenic factor *Sox9* and tenogenic scleraxis (*Scx*).^11^ Postnatally, these *Sox9*^+^/*Scx*^+^ progenitors undergo lineage bifurcation into terminal chondrocytes or tenocytes, whereas the cells in between subsequently differentiate into *Gli1*^+^ cells which will eventually establish mature fibrocartilage.^10,13^

Current understanding of enthesis progenitors remains incomplete due to insufficient integration of mechanobiological perspectives. Given that enthesis locates constantly in strong and variable mechanical environments.^14,15^ Consensus has been achieved that mechanical loading is crucial to the growth of enthesis fibrocartilage, and loss of mechanical stimulation eventually hinders the differentiation and mineralization of enthesis fibrochondrocytes.^14,16^ However, the single-cell level dynamics in enthesis development after unloading remain unclear. Herein, to solve the missing puzzle, we conducted comparative single-cell mapping of naturally loaded versus unloaded entheses. Our analysis revealed a previously unrecognized *Tnn*⁺ progenitor subpopulation serving as the cellular origin of fibrocartilage lineages. Functional validation demonstrated that tendon unloading directly modulates *Tnn*⁺ progenitor behavior, thereby elucidating mechanical regulation of enthesis morphogenesis at cellular resolution.

## Results

### Spatially resolved transcriptomic profiling to show distinct gene modules in embryonic tendon enthesis

**Fig. 1:**
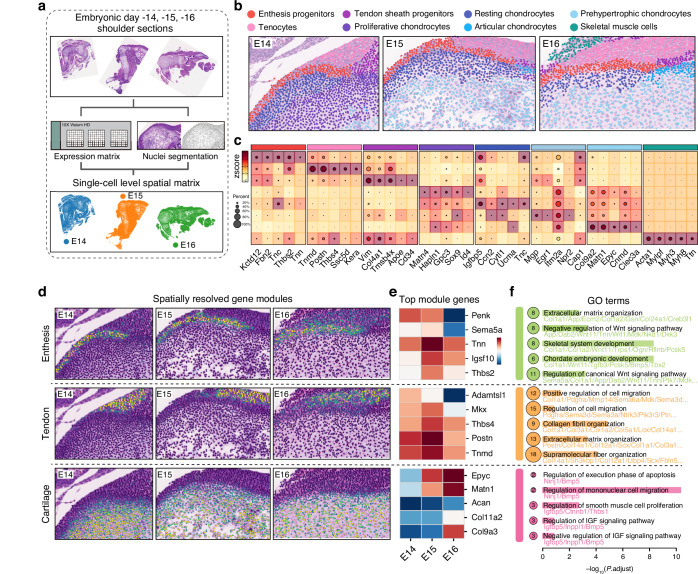
Spatially resolved transcriptomic profiling of mouse embryonic tendon enthesis. **a** Schematic overview of the spatial transcriptomics experimental design. **b** Uniform Manifold Approximation and Projection (UMAP) plot visualizing distinct cell clusters identified from supraspinatus tendon entheses at embryonic day 14 (E14), E15, and E16. Clusters include: enthesis progenitors (EP), tenocytes (TC), tendon sheath progenitors (TSP), proliferative chondrocytes (Prol.C), resting chondrocytes (RC), articular chondrocytes (AC), prehypertrophic chondrocytes (PreHC), and smooth muscle cells (SMC). **c** Heatmap to showing the top 5 differentially expressed genes (DEGs) defining each identified spatial cell cluster. **d**, **e** Spatial visualization of distinct gene modules (‘spatial topics’) identified using STAMP (**d**), and the expression levels of the corresponding highest-ranking genes from each associated gene module (**e**). **f** Enriched Gene Ontology (GO) terms associated with the corresponding highest-ranking genes from each gene module identified in (**d**, **e**)

To delineate spatiotemporal transcriptional dynamics during embryonic enthesis development, we implemented Visium HD spatial transcriptomic profiling of murine tendon entheses at embryonic day (E)14, E15, and E16. Nucleus segmentation was performed on high-resolution H&E images to map the spot-level gene expression matrices (Fig. 1a). Through rigorous region-of-interest (ROI) selection across the shoulder sections, we retained 4991 spatially resolved cellular spots within entheseal domains, achieving a median detection of 149 genes per segmented nucleus (Fig. S1a). BASS algorithm-driven spatial clustering^17^ revealed eight distinct cellular niches: enthesis progenitors (EP), tenocytes (TC), tendon sheath progenitors (TSP), proliferative chondrocytes (Prol.C), resting chondrocytes (RC), articular chondrocytes (AC), prehypertrophic chondrocytes (PC), and smooth muscle cells (SMC) (Fig. 1b). Region specific differential expression analysis identified compartment-specific signatures: EP domains were enriched for *Kctd12*, *Fbn2*, *Tnc*, *Thbs2*, and *Tnn*; adjacent TC regions expressed canonical tendon markers (*Tnmd*, *Postn*, *Thbs4*, *Kera*); RC populations exhibited cell cycle regulators (*Ccn2*, *Cytll1*) and cartilage-specific ECM components (*Igfbp5*, *Ucma*, *Tnc*) (Fig. 1c).

To better decipher the gene modules in different spatial domains, we performed interpretable spatially aware dimension reduction (STAMP)^18^ to get six spatially organized topics with associated gene modules ranked by their contribution to the topic (Fig. S1c). Visual examination of STAMP’s output showed well-defined spatial topics that corresponded to tendon, enthesis, and cartilage area (Fig. 1d, e). In genomic perspective, *Penk*, *Sema5a*, *Tnn*, *Igsf10*, and *Thbs2* were highest relative to the gene topic exclusive to enthesis (Fig. S1d). Spatial mapping confirmed exclusive *Tnn*/*Penk* localization within entheseal zones (Fig. 1e). Gene ontology analysis revealed EP-specific enrichment in extracellular matrix organization, skeletal morphogenesis, chordate embryonic development, and Wnt signaling pathways, which was distinct from transcriptional programs in tendons or articular chondrocytes (Fig. 1f).

### Single-cell transcriptomic profiling and lineage tracing identified *Tnn*⁺ progenitors as cellular origins of enthesis fibrocartilage

**Fig. 2:**
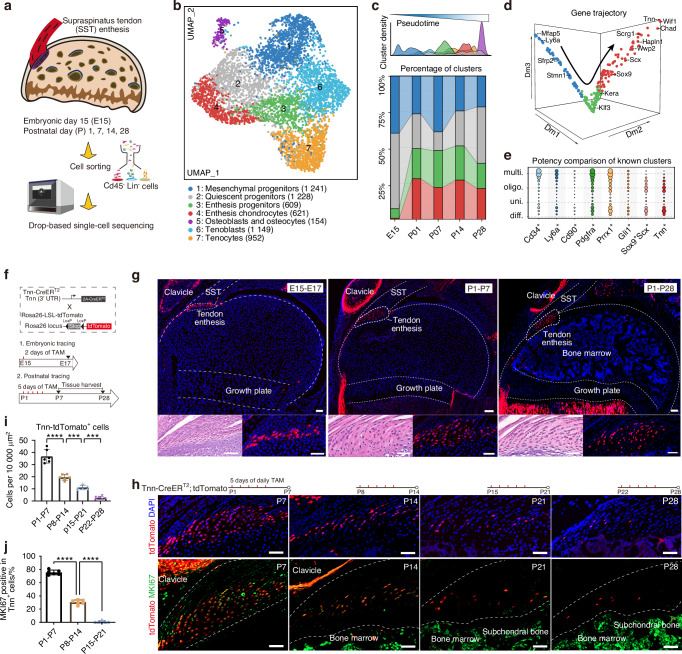
Single-cell transcriptomic mapping and in vivo lineage tracing identify *Tnn*^+^ enthesis progenitors. **a** Schematic overview of the scRNA-seq experiment. **b** UMAP plot visualizing integrated cell clusters (mesenchymal progenitors, Quiescent progenitors, enthesis progenitors and chondrocytes, osteoblasts and osteocytes, enthesoblasts, tenocytes) from developing entheses at embryonic day 15 (E15), postnatal day 1 (P1), P7, P14, and P28. **c** Percentages of each cell clusters across pseudotime and at distinct developmental timepoints. **d** Diffusion map visualizing gene expressions along the chondrogenic differentiation trajectory. **e** Feature plots comparing the differentiation potential (Cytotrace score) of *Tnn*^+^ cells versus other well-known stem/progenitor cells. **f** Schematic diagram of tamoxifen administration in *Tnn*^+^ lineage tracing. To label cells started from E15, Tnn-Cre^ERT2^; tdTomato pregnant dams received two consecutive daily tamoxifen (75 mg/kg body weight), and embryos were harvested at E17. To label cells from P1, newborn pups received five consecutive daily tamoxifen (50 mg/kg body weight) injections from P1 to P5. **g** Representative fluorescence and H&E-stained images of the supraspinatus tendon enthesis harvested from Tnn-Cre^ERT2^; tdTomato mice. Scale bars = 100 μm. At least 3 mice were used and the representative images were shown. **h** Representative images of pulse-traced *Tnn*^+^ cells and their progeny cells, and immunofluorescent images of co-stained Ki67 at different time windows (*n* = 6 mice per group). Scale bars = 100 μm. **i** Quantitative comparisons of pulse traced Tnn-tdTomato^+^ at different time windows (*n* = 6 mice per group). Two-way ANOVA and Tukey post-hoc test. Data presented as mean ± SEM. ***P < 0.001, ****P < 0.000 1. **j** Quantitative comparisons of KI67 positive rates in Tnn-tdTomato^+^ cells at different time windows (*n* = 6 mice per group). Two-way ANOVA and Tukey post-hoc test. Data presented as mean ± SEM. ****P < 0.000 1

To resolve the cellular heterogeneity underlying enthesis morphogenesis, we constructed a single-cell transcriptomic atlas starting from embryonic (E15) to postnatal (P28) developmental stages (Fig. 2a). After quality control, data integration and elimination of blood cells, endothelial cells, immune cells, and growth plate chondrocytes as previous reported^12^ (Fig. S2), we finally got high-quality transcriptomic data from 5 954 single cells (Fig. 2b). Cell clusters were manually annotated according to their canonical marker expressions, including mesenchymal progenitors (*Ly6a*, *Cd34*), enthesis chondrocytes (*Col2a1*, *Chad*), tenoblasts (*S100a4*, *Tppp3*), and tenocytes (*Tnmd*, *Kera*).^8,9,11,19^ We identified cluster 3 as enthesis progenitors based on three key lines of evidence. First, these cells showed a clear commitment to the fibrocartilage lineage by co-expressing chondrogenic markers like *Acan* and *Hapln1* alongside genes for fibrous Type I collagen (*Col1a1*). Second, their progenitor status was confirmed by CytoTRACE analysis (Fig. S3e), which revealed a significantly higher differentiation potential compared to the mature enthesis chondrocytes (Cluster 4). Finally, this population was defined by a dominant hypoxic and glycolytic metabolic signature with high expression of *Ndufa4l2*, *Pkm*, and *Ldha*, indicating their adaptation to the low-oxygen niche, which has been regarded as the metabolic onset of chondrocyte hypertrophy.^20^

Notably, we found cells from cluster 2 were enriched for markers of both quiescence (*Cdkn1c*) and early chondrogenic potential (*Itm2a*, *Dlk1*, *Mdk*), rather than markers of active proliferation. Furtherly, we found *Sfrp2* (Wnt antagonist) and *Dkk2* (another Wnt/beta-catenin signaling inhibitor) were present in cluster 2, along with *Igfbp4* (IGF antagonist). Suggestive of these cells were actively inhibiting signaling pathways that would push themself towards proliferation and differentiation, kept them in a ‘poised’ state. Therefore, to more accurately reflect its biological state, we renamed this population as Quiescent progenitors.

The cell number and pseudotime analysis of these clusters identified tenocytes and enthesis chondrocytes as terminal cells which belonging to tenogenic and chondrogenic lineage, respectively. We observed a notable decrease in the proportion of quiescent progenitors between P1 and P7 postnatally. This decrease coincides with the establishment of the enthesis progenitor and chondrocytes population. The period after this (P14 to P28) shows more stability, suggesting that once the main ‘wave’ of differentiation has occurred to form the fibrocartilage, and the peak in the quiescent progenitor population at this time reflects a maturing tissue preparing for long-term maturation and homeostasis (Fig. 2c).

To deconvolve the chondrogenic differentiation pathway, GeneTrajectory^21^ and CellRank2^22^ pseudotime were calculated to reconstruct the trajectory of enthesis chondrocyte differentiation and to calculate its top driving genes (Fig. 2d and Fig. S3g, h), this dual-method approach identified *Tnn* as a principal driver gene along the chondrogenic trajectory, with ascending expression correlating with lineage commitment. We performed comparative analysis of well-known skeletal progenitors (*Cd34*^+^, *Cd90*^+^, *Ly6a*^+^, *Pdgfra*^+^, *Prrx1*^+^, *Gli1*^+^, *Sox9*^+^*Scx*^+^ enthesis precursors)^7,11,23–25^ with *Tnn*^+^ populations. And we compared their differentiation potentials with *Tnn*^+^ cells by using Cytotrace2 method,^26^ it showed that although the differentiation ability of *Tnn*^+^ cells was inferior to those canonical progenitors, but it was consistent with the enthesis *Gli1*^+^ and *Sox9*^+^*Scx*^+^ precursors (Fig. 2e). Indicated that *Tnn*^+^ cells may not possess the multi-lineage differentiation potential, and their differentiation trajectory is more fixed toward fibrochondrogenesis.

To further elucidate the temporal and spatial distribution of *Tnn*^+^ cells during enthesis development, we generated Tnn-Cre^ERT2^; tdTomato lineage-tracing mice. Our results showed that as early as embryonic day 15 (E15), a distinct population of *Tnn*^+^ enthesis progenitors appeared in the enthesis area, separate from tenocytes and epiphyseal chondrocytes (Fig. 2f–j). Postnatally, the distribution of *Tnn*^+^ enthesis progenitors (pulse-trace started from P1) and their progeny cells in tendon entheses remained highly consistent when observed at P7, P14, and P28 (Fig. 2g) and predominantly located at tendon entheses, cortical bone, and metaphyseal areas. Collectively, the in vivo distribution of *Tnn*^+^ cells was primarily concentrated in mineralization-associated regions, such as the sub-clavicular fibrocartilage and the fibrocartilaginous layer at the bone-tendon interface. Conversely, *Tnn*^+^ cells were not detected in articular cartilage or the primitive chondrocytes of the developing humeral head.

We then compared the expression patterns of *Tnn* and *Gli1* in single cell datasets. *Tnn*^+^ and *Gli1*^+^ cells were predominantly located within the same progenitor and enthesis populations. This expression overlap (Fig. S4a, b) suggests they originate from a similar cellular niche and participate in the same developmental process. Gene Ontology (GO) analysis of *Tnn*^+^ and *Gli1*^+^ cells similar biological processes crucial for enthesis formation, such as ossification, bone mineralization, and extracellular matrix organization. This shared functional signature indicates that both populations are actively contributing to the formation of the fibrocartilaginous enthesis. The most critical difference is their opposing quantity trends as showed in Fig. S4c. The *Tnn*^+^ population is dominant in the early postnatal period, with 90.8% of cells expressing *Tnn* at P1 then undergoes a progressive decline to 56.6% by P7 and just 35% by P28 (Fig. S4d). In contrast, the *Gli1*^+^ population starts as a minority (23.3% at P1) and progressively expands, ultimately becoming the majority population by P28 (53.7%).

### *Tnn*^+^ cells in neonatal enthesis exhibited fibrochondrogenic potency

**Fig. 3:**
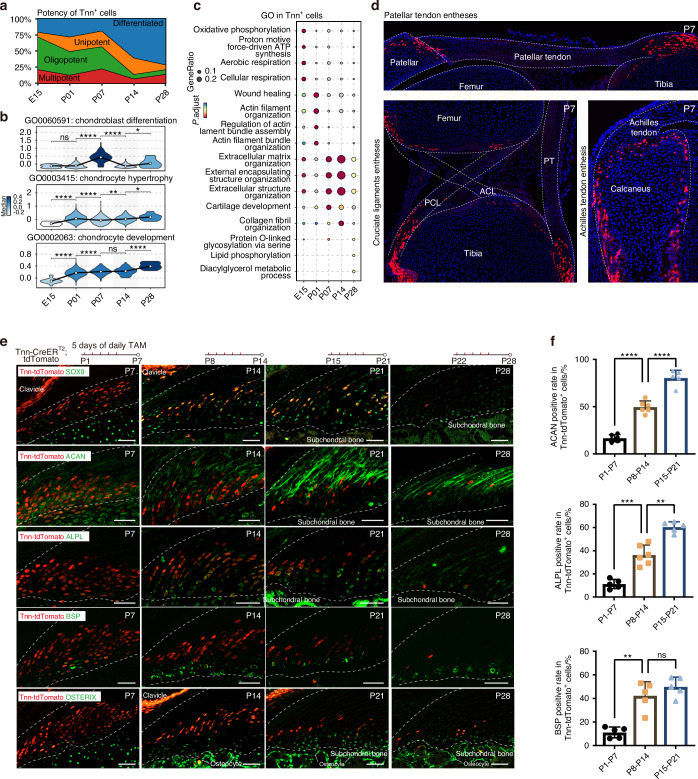
*Tnn*^+^ enthesis progenitors in enthesis development exhibit fibrochondrogenic potency. **a** Stacked area chart comparing the differentiation potential (Cytotrace score) of *Tnn*^+^ progenitors at different developmental time points. **b** Expression scores of genes involved in chondroblast differentiation, chondrocyte hypertrophy, and chondrocyte development in *Tnn*^+^ cells at various time points. **c** Dot plots showing the most enriched GO terms in *Tnn*^+^ cells across different time points. **d** Representative tdTomato labeling images showing abundant distribution of *Tnn*^+^ cells at the attachment sites of cruciate ligaments, patellar tendon entheses, and Achilles tendon entheses at P7. Tnn-Cre^ERT2^; tdTomato newborn pups received five consecutive daily intraperitoneal injections of tamoxifen (50 mg/kg body weight) since P1. **e** Representative tdTomato labeling images of pulse-traced *Tnn*^+^ cells and their progeny cells co-stained with immunofluorescence markers, including early enthesis differentiation (SOX9), chondrocyte maturation (ACAN), biomineralization (IBSP, ALPL), and osteoblast specific marker (OSTERIX). Five consecutive tamoxifen injection was administered from P1-P5, P8-P12, P15-P19, and P22-P26, with tissues harvested for analysis on P7, P14, P21, and P28, respectively. Scale bars = 50 μm. **f** Quantification of the percentage of tdTomato labeled cells that co-express ACAN, ALPL, and BSP at each time window (*n* = 5–6 mice per group). One-way ANOVA and Tukey post-hoc test. Data presented as mean ± SEM. **P < 0.01, ***P < 0.001, ****P < 0.000 1

Cytotrace2 analysis was performed to investigate the cellular potency and developmental potential of *Tnn*^+^ cells at different developmental stages. The results showed that most *Tnn*^+^ cells exhibited oligopotency during embryonic and neonatal stages, with this characteristic diminishing as age increased (Fig. 3a). Furthermore, *Tnn*^+^ cells isolated from the tendon entheses at P1 showed robust colony-forming ability in vitro (Fig. S6b). First-passage *Tnn*^+^ cells cultured in vitro were subsequently analyzed for the expression of stem cell-associated surface markers, and the results showed that over 90% of these *Tnn*^+^ cells expressed *CD29* and *CD44* (Fig. S6c), indicating that *Tnn*^+^ cells derived from the tendon enthesis at postnatal day 1 possess significant clonogenic properties.

In terms of the biological function of *Tnn*^+^ cells in enthesis development, GO enrichment scores showed that chondroblast differentiation was significantly upregulated in *Tnn*^+^ cells at P7, and the genes associated with chondrocyte hypertrophy and differentiation showed progressively higher expression levels in *Tnn*^+^ cells with increasing age (Fig. 3b). Functional enrichment analysis of *Tnn*^+^ cells at P7, P14, and P28 revealed their increasingly involvement in processes such as chondrocyte differentiation, extracellular matrix synthesis, proteoglycan synthesis, and collagen fiber organization (Fig. 3c).

Given that the spatial distribution of *Tnn*^+^ cells across the body had not been previously reported, we investigated their presence in major organs throughout the body. The results showed that *Tnn*^+^ cells were not detected in heart, liver, or brain tissues. However, they were abundantly present in the small bronchi of the lung (P14), the fontanelle (P14), and at the tendon attachment sites, including the anterior and posterior cruciate ligaments (P7), patellar tendon entheses (P7), and Achilles tendon entheses (P7) (Figs. 3d, S6a). Notably, our results showed that *Tnn* marked a broader of cell types involved in not only enthesis development but also osteogenesis tissues within periosteum of the clavicle and humerus (Fig. S5). Suggesting that the Tnn-positive cell population in body is not a single, homogeneous cluster but including at least two major developmental trajectories: One is osteogenic lineage (Cluster 5) defined by the expression of *Runx2* and *Sp7*, as well as periosteum markers (*Prrx1* and *Ctsk*). And another chondrogenic/fibrocartilaginous lineage (Cluster 3 and 4) defined by the expression of *Col2a1* and *Scrg1* and characteristic of the tendon enthesis.

At tendon entheses, *Tnn*^+^ cells were preferentially located within the fibrocartilage area and were not detected in the subchondral bone layer. To further validate the differentiate potential of *Tnn*^+^ cells in different time points, we co-stained *Tnn*^+^ cells with various markers, including early chondrocyte differentiation (SOX9), chondrocyte maturation (ACAN), biomineralization (ALPL, BSP), and osteoblast specific marker (OSTERIX) (Fig. 3e). The results showed that *Tnn*^+^ cells exhibited more significant co-localization with the chondrogenic marker (SOX9 and ACAN) rather than with the osteogenic marker OSTERIX. Additionally, a substantial proportion of entheseal *Tnn*^+^ cells began to express ALPL at later postnatal stages (P8-P21), consistent with their differentiation into a mineralizing phenotype (Fig. 3f). These results indicate that *Tnn*^+^ cells and their progeny at tendon entheses are primarily involved in the development and maturation of the fibrocartilage.

### Ablating the *Tnn*^+^ enthesis progenitors impairs fibrocartilage development

**Fig. 4:**
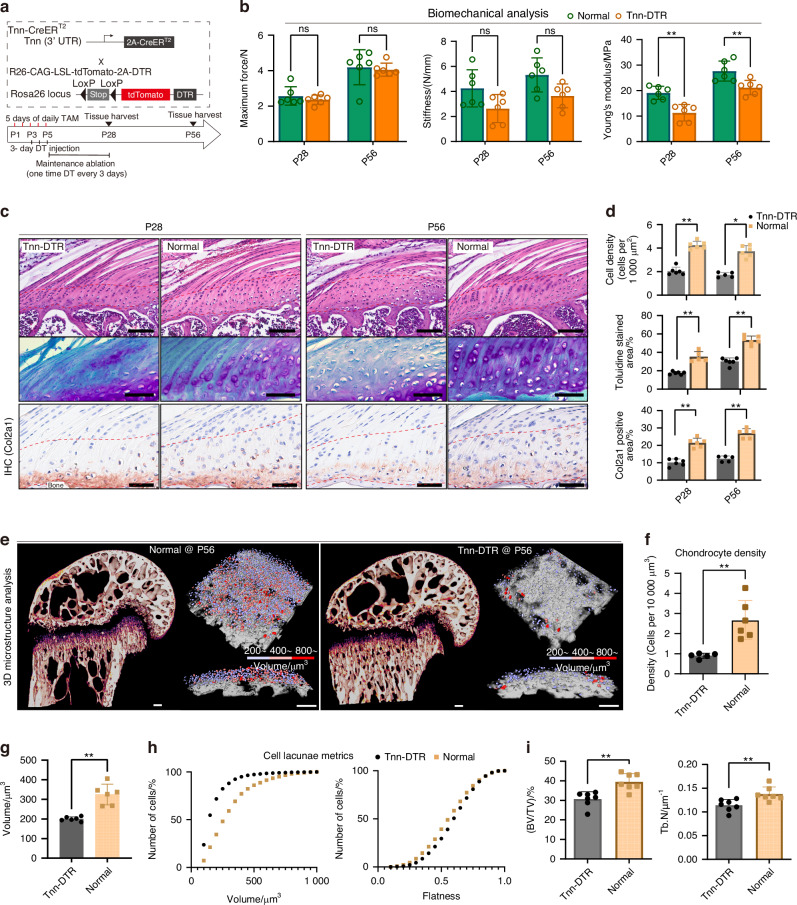
Ablation of *Tnn*^+^ enthesis progenitors impairs fibrocartilage development and biomechanical properties. **a** Schematic of the experimental design for *Tnn*^+^ cell ablation. Newborn Tnn-Cre^ERT2^; iDTR mice received five consecutive injections of tamoxifen (50 mg/kg body weight) starting at P1. When two days after the first tamoxifen injection (at P3), mice received 3 days of daily injection of Diphtheria Toxin (DT; 5 ng/g body weight), followed by regular DT injection every 3 days until the age of 4 weeks. **b** Biomechanical properties (stiffness, Young’s modulus, maximum load) of tendon entheses from control and Tnn-DTR groups at specified postnatal time points (*n* = 6 mice per group). Mann-Whitney U test. Data presented as mean ± SEM. **P < 0.01. **c**, **d** Representative histological images (H&E, Toluidine Blue-Fast Green, and immunohistochemistry for COL2A1) of SST entheses from control and Tnn-DTR groups. (*n* = 5–6 mice per group). Scale bars = 100 μm. Mann-Whitney U test. Data presented as mean ± SEM. *P < 0.05, **P < 0.01. **e**, **f** Representative 3D Synchrotron Radiation micro-Computed Tomography (SR-μCT) sagittal images (**e**) of control and Tnn-DTR tendon entheses, and corresponding 3D visualizations of fibrocartilage cells (**f**) (*n* = 6 mice per group). Scale bars = 100 μm. Student’s *t*-test. Data presented as mean ± SEM. **P < 0.01. **g** Comparison of fibrocartilage cell lacunar volume between control (normal) and Tnn-DTR (*Tnn*^+^ cell-ablated) groups (*n* = 6 mice per group^)^. Student’s *t*-test. Data presented as mean ± SEM. **P < 0.01. **h** Frequency distributions of volume and flatness, between control (normal) and Tnn-DTR (*Tnn*^+^ cell-ablated) groups. **i** Quantitative comparison of subchondral bone morphometric parameters, including bone volume fraction (BV/TV) and trabecular number (Tb.N), between control and Tnn-DTR groups (*n* = 6 mice per group). Student’s *t*-test. Data presented as mean ± SEM. **P < 0.01

To determine the functional role of *Tnn*^+^ cells in enthesis development in vivo, we generated Tnn-Cre^ERT2^; iDTR mice for inducible ablation of *Tnn*^+^ cells. Rotator cuff specimens were harvested from control (Normal) and Tnn^+^ cell-ablated (Tnn-DTR) groups at postnatal day 28 (P28) and P56 for analysis (Fig. 4a). Biomechanical testing revealed that Young’s modulus of the tendon entheses at both 4 weeks (P28) and 8 weeks (P56) postnatally were significantly decreased in the Tnn-DTR group compared to the control group (*P* < 0.01) (Fig. 4b).

Histological analysis demonstrated that at both P28 and P56, the maturity of enthesis fibrocartilage in the Tnn-DTR group was significantly impaired compared to the control group (Fig. 4c, d). This impairment was characterized by the absence of typical layered structures and decreased density of fibrochondrocytes. Additionally, the area of Toluidine Blue staining (at P28: 17.5% vs. 35.3% in controls, *P* < 0.01; at P56: 30.0% vs. 53.5% in controls, *P* < 0.01) and COL2A1 IHC staining (at P28: 10.7% vs. 18.3% in controls, *P* < 0.01; at P56: 12.3% vs. 25.5% in controls, *P* < 0.01) were significantly reduced in the Tnn-DTR group, indicating diminished extracellular matrix synthesis within the fibrocartilage layer.

Morphological analysis of fibrocartilage revealed that compared to normal tendon entheses, those from the *Tnn*^+^ cell-ablated (Tnn-DTR) group exhibited significant reductions in both and fibrocartilage cell density (at 8 weeks: 0.89 vs. 2.67 cells/10⁴ μm³, respectively; *P* < 0.01) (Fig. 4f). Three-dimensional analysis of fibrocartilage cell morphology showed that cell volume, surface area, anisotropy, and flatness were all significantly reduced in the Tnn-DTR group compared to controls. This was consistent with size distribution analyses indicating overall smaller fibrocartilage cells following the ablation of *Tnn*^+^ enthesis progenitors (Fig. 4g, h). Bone volume fraction (BV/TV) was significantly decreased in the Tnn-DTR group compared to the control group (*P* < 0.01). Moreover, trabecular number (Tb.N) in the subchondral bone of the Tnn-DTR group was lower than that in the control group (Fig. 4i). Further examination of fibrocartilage calcification at the rotator cuff tendon-bone interface at 8 weeks postnatally was performed using transmission electron microscopy (TEM). TEM analysis revealed a significant reduction in calcium-based mineralization products within the fibrocartilage of Tnn-DTR entheses compared to controls. Notably, the calcium-to-phosphorus ratio was significantly lower in the Tnn-DTR group (Fig. S7a, b).

Furthermore, qRT-PCR analysis revealed that, compared to control entheses, the expression of key genes involved in collagen synthesis (*Col1a1*, *Col2a1*), chondrocyte hypertrophic differentiation (*Clec3a*, *Mef2c*), and mineralization (*Ibsp*, *Spp1*, *Alpl*, *Sost*) was variably reduced in the Tnn-DTR group at both 4 and 8 weeks postnatally (Fig. S7c).

### Tendon unloading during development induces enthesis hypoplasia

**Fig. 5:**
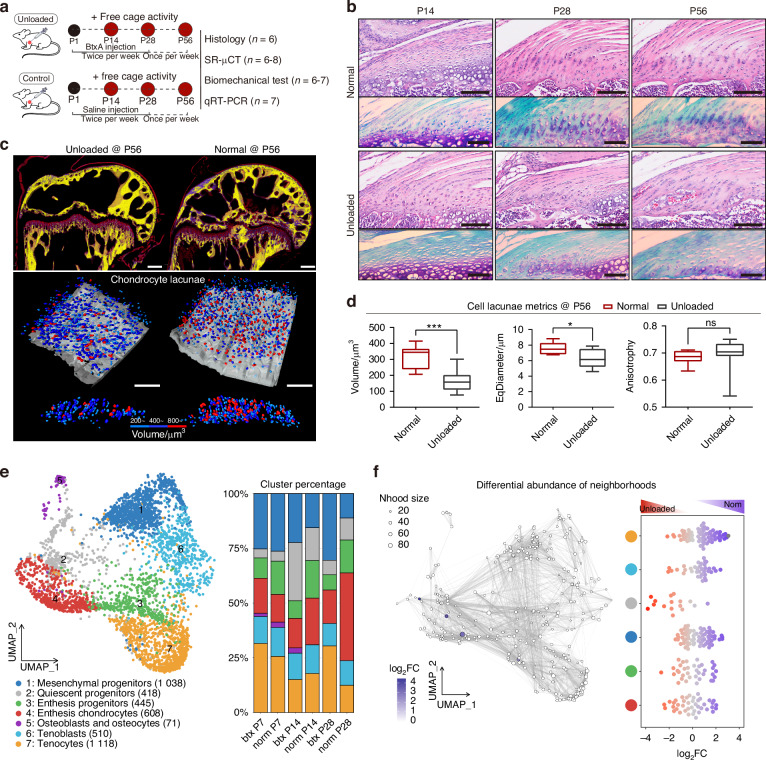
Tendon unloading via botulinum toxin (BTX) injection induces enthesis hypoplasia during postnatal development. **a** Schematic illustrating BTX-treated and control groups and subsequent experiments. 0.2U of BtxA was injected twice per week in the left supraspinatus muscle from birth until 4 weeks and then once per week through sacrifice at P56. The contralateral supraspinatus muscles were injected with the same amount of saline and served as controls. **b** Representative H&E staining and Toluidine Blue-Fast Green staining of supraspinatus tendon entheses from C57BL/6 J mice in the BTX-treated and control groups (*n* = 6 mice per group). Scale bars = 100 μm. **c** Representative 3D Synchrotron Radiation micro-Computed Tomography (SR-μCT) sagittal images of SST entheses from BTX-treated and control groups, with corresponding 3D visualizations of fibrocartilage cells. Scale bars = 100 μm. **d** Comparison of fibrocartilage cell lacunar metrics, including volume, equivalent diameter, and anisotropy, from BTX-treated and control groups (*n* = 8 mice per group). Student’s *t*-test. Data presented as mean ± SEM. *P < 0.05, ***P < 0.001. **e** UMAP plot (left) and bar plot (right) showing cell cluster distribution and relative percentage from integrated scRNA-seq data of control and BTX-treated entheses at postnatal day 7 (P7), P14, and P28. **f** Milo differential abundance analysis of enthesis cell populations. Left: neighborhood graph (nodes colored by log₂FC; size proportional to cell count). Right: neighborhood abundance by cell type; blue and red dots indicate significantly decreased and increased abundance, respectively (SpatialFDR < 0.1)

To investigate the effect of mechanical loading on fibrocartilage formation, 0.2U of Botulinum toxin A (BtxA) was injected twice per week in the left supraspinatus muscle from birth until 4 weeks and then once per week through sacrifice at P56 (Fig. 5a). Histological analysis (H&E and Toluidine Blue-Fast Green staining) revealed that the overall maturity of enthesis fibrocartilage was impaired in the unloaded (BTX) group evidenced by the absence of tidemarks and significantly decreased toluidine blue stained areas in unloaded entheses (Figs. 5b, S8a). These alterations in fibrocartilage phenotype were further supported by changes in 3D fibrocartilage cell morphology changes, including the volume and equivalent diameter of fibrochondrocyte lacunae were significantly decreased in the unloaded group, indicating that fibrochondrocytes became smaller and more circular following tendon unloading (Fig. 5c, d). Biomechanical testing showed that at 8 weeks postnatally, the maximum force and stress of entheses in the unloaded group were significantly lower than those in the control group (*P* < 0.001). However, no significant differences were observed at 4 weeks postnatally (Fig. S8c).

Single-cell RNA sequencing (scRNA-seq) was employed to compare cellular composition changes between entheses from control and unloaded groups at 7, 14, and 28 days postnatally. Consistent with histological findings, the percentage of enthesis chondrocytes in the unloaded group was significantly lower than in the control group at P14 and P28 (Fig. 5e). Differential abundance testing using Milo confirmed a notable decrease in mesenchymal progenitors and enthesis chondrocytes in the BTX group, contrasting with an increase in enthesis progenitors observed in the control group over pseudotime (Fig. 5f).

**Fig. 6:**
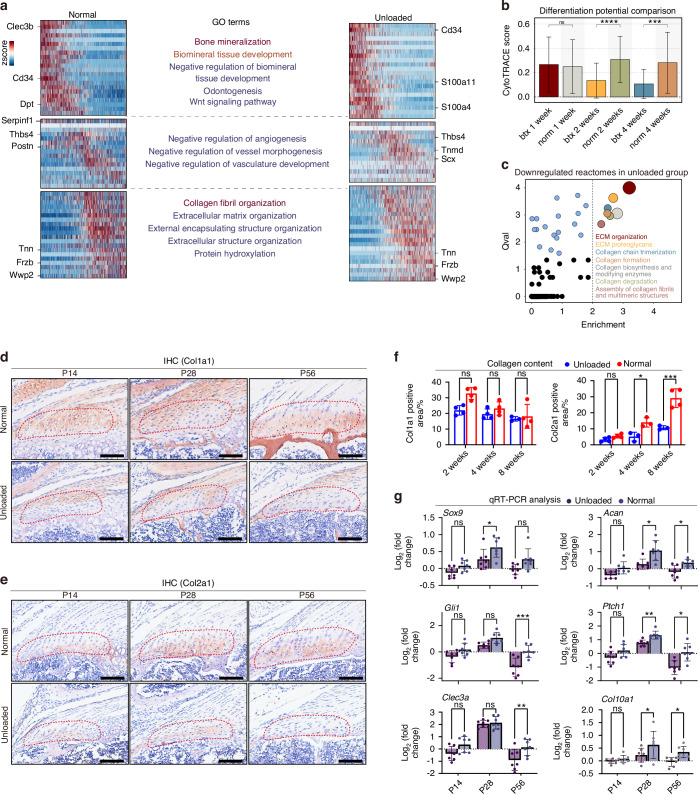
Tendon unloading impairs enthesis chondrogenesis during postnatal development. **a** Heatmaps showing dynamic changes in the expression levels of significant genes along the chondrogenic pseudotime trajectory, comparing between control and BTX-treated groups. Enriched gene ontology terms for different gene expression patterns are shown in the middle. **b** Comparison of differentiation potential (Cytotrace score) of enthesis chondrocytes between control (normal) and BTX-treated (unloaded) groups at specified time points. **c** Single-Cell Pathway Analysis (SCPA) identifying the most significantly downregulated reactome pathways in enthesis chondrocytes from the unloaded group compared to controls. Representative IHC images (**d**, **e**) and corresponding quantification (**f**) for COL1A1 and COL2A1 protein expression from control and unloaded (BTX-treated) groups (*n* = 3–4 mice per group). Scale bars = 100 μm. Multiple unpaired *t*-test. Data presented as mean ± SEM. *P < 0.01, ***P < 0.001. **g** Relative mRNA expression levels of genes related to chondrocyte development (*Sox9*, *Acan*), collagen synthesis (*Col1a1*, *Col2a1*), chondrocyte hypertrophy (*Clec3a*, *Col10a1*), and the Hedgehog signaling pathway (*Gli1*, *Ptch1*) from control and BTX groups (*n* = 7–8 mice per group). Two-way ANOVA and Tukey post-hoc test. Data presented as mean ± SEM. *P < 0.05, **P < 0.01, ***P < 0.001

To compare gene expression dynamics during enthesis chondrogenesis between BTX and control groups, we analyzed genes with significant expression changes along the chondrogenic trajectory and categorized them by expression pattern (Fig. 6a). Gene Ontology (GO) analysis revealed that chondrogenic trajectories from both BTX and control groups shared similar GO term patterns. However, the overall expression levels of genes within these GO terms related to bone mineralization, biomineral tissue development, collagen fiber organization, and ECM organization were downregulated in the BTX group.

Regarding differentiation potential, enthesis cells in the unloaded group exhibited significantly lower potential compared to those in control entheses at postnatal week 2 (Fig. 6b). Single-cell pathway analysis (SCPA) was utilized to identify significantly downregulated functional gene sets in fibrocartilage cells from the unloaded group. This analysis revealed that the expression of genes related to collagen formation and matrix synthesis was significantly decreased in fibrocartilage cells from unloaded entheses compared to controls (Fig. 6c). Furthermore, expression scores for genes involved in chondrocyte development, hypertrophy, and the Hedgehog signaling pathway were also significantly reduced in the unloaded group (Fig. S8d).

Type I collagen (COL1A1) is predominantly found in tendon soft tissues and bone, forming a critical component of their extracellular matrix, but is typically expressed at low levels in cartilage. Immunohistochemistry showed no significant difference in COL1A1 expression was observed between the two groups at 2, 4, and 8 weeks postnatally. Type II collagen (COL2A1) is cartilage-specific, and its expression in the tendon enthesis correlates with the degree of fibrocartilage mineralization. Our results demonstrated that at both 4 and 8 weeks postnatally, COL2A1 expression in the unloaded group was significantly lower than in the control group (at 4 weeks: 5.11% vs. 14.01%, respectively, *P* < 0.01; at 8 weeks: 10.68% vs. 29.11%, respectively, *P* < 0.001) (Fig. 6d–f).

qRT-PCR was used to measure gene expression in entheses from control and unloaded groups, focusing on genes involved in chondrocyte development (*Sox9*, *Acan*), collagen synthesis (*Col1a1*, *Col2a1*), chondrocyte hypertrophy (*Clec3a*, *Col10a1*), and the hedgehog signaling pathway (*Gli1*, *Ptch1*). The results showed that the expression of these genes was variably reduced at different postnatal time points in the unloaded group, suggestive of downregulated chondrocyte development, differentiation, and mineralization following the removal of tendon loading (Fig. 6g).

### Tendon unloading reduces the number and chondrogenic potential of *Tnn*^+^ enthesis progenitors

**Fig. 7:**
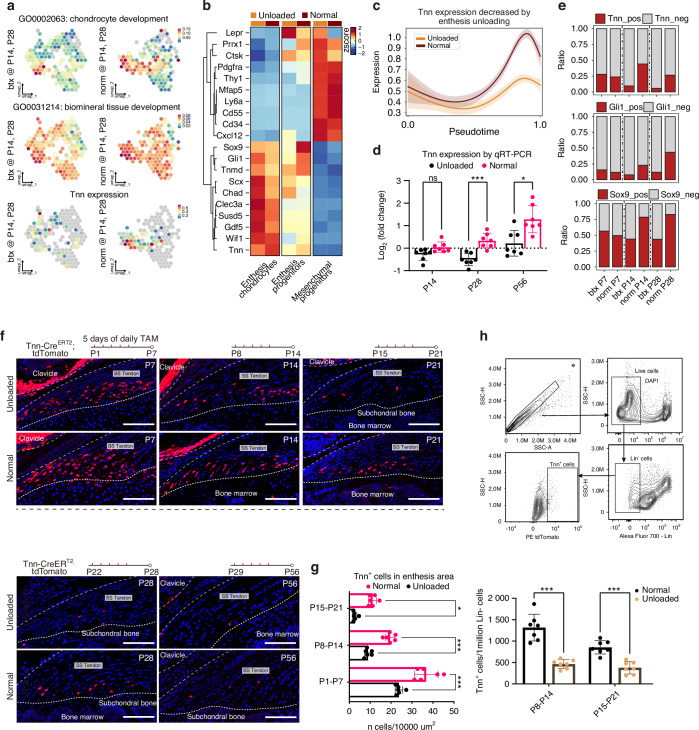
Reduced mechanical loading via BTX treatment decreases the number and chondrogenic potential of Tnn^+^ enthesis progenitors. **a** Feature plots showing the expression of representative genes associated with chondrocyte development, biomineral tissue development, and *Tnn*, comparing between control (normal) and BTX-treated (unloaded) groups. **b** Heatmap showing the expression of genes related to stemness, chondrogenesis, and extracellular matrix (ECM) organization within chondrogenic lineage clusters, comparing control and BTX-treated groups. **c** Line plots showing decreased *Tnn* expression levels along the chondrogenic pseudotime trajectory in the BTX-treated group compared to controls. **d** qRT-PCR analysis demonstrating significantly reduced Tnn mRNA expression levels in entheses from the BTX-treated group compared to controls (*n* = 6–7 mice per group). Two-way ANOVA and Tukey post-hoc test. Data presented as mean ± SEM. *P < 0.05, ***P < 0.001. **e** Comparison of the proportion of cells expressing known stem cell markers between control and BTX-treated groups, based on scRNA-seq data. **f** Representative lineage tracing images of *Tnn*^+^ progenitors at entheses from BTX and control groups. Tnn-Cre^ERT2^; tdTomato mice were subjected to enthesis unloading (BTX) or saline treatment (Control). Five consecutive tamoxifen injection was administered from P1-P5, P8-P12, P15-P19, and P22-P26, with tissues harvested for analysis on P7, P14, P21, and P28, respectively. Scale bars = 100 μm. **g** Quantification of *Tnn*^+^ enthesis progenitor numbers from IF images (as shown in F) in control and BTX-treated groups at various postnatal time points (*n* = 6 mice per group). Two-way ANOVA and Tukey post-hoc test. Data presented as mean ± SEM. *P < 0.05, ***P < 0.001. **h** Flow cytometry (FACS) analysis comparing the absolute number or percentage of *Tnn*^+^ enthesis progenitors between control and BTX-treated groups (*n* = 7 mice per group). Two-way ANOVA and Tukey post-hoc test. Data presented as mean ± SEM. ***P < 0.001

We compared the expression profiles of chondrogenic genes between BTX treated (unloaded) and control (normal) groups and found the genes associated with chondrocyte development and biomineral tissue development were significantly downregulated in the BTX group (Fig. 7a). Similarly, the expression of genes related to stemness, chondrocyte differentiation, and ECM organization was reduced within chondrogenic lineage clusters (mesenchymal progenitors, enthesis progenitors, and enthesis chondrocytes) in the unloaded group (Fig. 7b). Notably, the expression level of the chondrogenic driving gene *Tnn* was also downregulated in the BTX group (Fig. 7c, d). To investigate whether a reduction in progenitor cell number contributed to the impaired fibrocartilage differentiation following unloading, we compared the proportion of cells expressing known stem cell markers such as *Gli1*, *Pdgfra*, *Prrx1*, *Lepr*, *Ctsk*, *Ly6a*, *Cd34*, and *Thy1*^7,10,23,25,27,28^ between control and unloaded groups. The results indicated that the number of *Tnn*^+^, *Clec3a*^+^, and *Gli1*^+^ cells was significantly decreased in unloaded entheses, while other stem cell markers did not exhibit a clear correlation with mechanical unloading (Figs. 7e, S9a).

Consistent with scRNA-seq findings, both the proportion of *Tnn*^+^ cells and their differentiation potential were significantly lower in unloaded entheses compared to controls (Fig. 7d, e). To validate these findings and track *Tnn*^+^ cell dynamics, *Tnn*^+^ enthesis progenitors were quantified at various postnatal intervals (P1-P7, P8-P14, P15-P21, P22-P28, and P29-P56). *Tnn*^+^ cells were most abundant during early postnatal development (1–3 weeks), with their numbers gradually decreasing with age. Particularly during early development (within 2 weeks postnatally), the number of *Tnn*^+^ cells in unloaded entheses was significantly lower than that in control entheses (P1-P7: 23.7 vs. 36.8 cells/10⁴ μm², respectively, *P* < 0.001; P8-P14: 8.9 vs. 19.5 cells/10⁴ μm², respectively, *P* < 0.001). By the postnatal week 4, *Tnn*^+^ cells were scarcely detectable in either control or unloaded groups (Fig. 7f). Furthermore, absolute numbers of *Tnn*^+^ cells, quantified by flow cytometry, were significantly decreased in the unloaded group compared to controls (P8-P14: 463 vs. 1 315 cells/10⁶ Lin^-^ cells, respectively, *P* < 0.001; P21-P28: 377 vs. 854 cells/10⁶ Lin^-^ cells, respectively, *P* < 0.001) (Fig. 7g).

Functional assessment revealed that *Tnn*^+^ cells from unloaded (BTX) entheses exhibited reduced differentiation potency scores and decreased expression of genes associated with chondrocyte development and biomineral tissue development (Figs. 7e, S9b, c). Considering that *Tnn* is a key driver gene in enthesis fibrocartilage development and that both the proportion of *Tnn*^+^ cells and their chondrogenic potential were significantly reduced following unloading, these findings collectively suggest that *Tnn*^+^ cells represent a mechanically responsive population of enthesis precursors.

Previous studies have shown that chondrocytes sense mechanical stimuli through various mechanisms, including ion channels (Piezo1 and Piezo2,^29^ as well as transient receptor potential TRP channels^30^), integrin family,^31^ microfilaments and microtubules,^32^ and primary cilia.^15^ In light of this, we scrutinized the expression profiles of these reported mechanosensitive channels or proteins in *Tnn*^+^ enthesis progenitors. Considering that *Tnn*^+^ cells mainly appeared in the early stages of enthesis development (from 1 day to 3 weeks after birth) and hardly observed after 4 weeks of birth, only the single-cell data from 1 and 2 weeks of development were statistically analyzed. The results showed that in the gene expression of ion channels (Piezo and TRP families) and integrins (ITG family), the positive proportion of cells for PIEZO1, TRPV4, ITGAV, ITGA5 in *Tnn*^+^ enthesis progenitors from unloaded groups were most significantly reduced (Fig. S13a), whereas no significant difference in the expression of other signaling molecule was observed (data not shown). Furthermore, comparison of differentiation potential revealed that Tnn^+^ cells expressing these mechanosensitive ion channels (PIEZO1, TRPV4, ITGA5, ITGAV) exhibited higher chondrogenic potency than Tnn^+^ cells negative of these mechanosensitive markers (Fig. S13b).

We next validated the expression of *Piezo1* and *Trpv4* in *Tnn*^+^ cells using immunofluorescence co-staining. At P7 and P14, the proportion of *Tnn*^+^ cells co-expressing Piezo1 or Trpv4 was significantly reduced following tendon unloading (*Piezo1* at P7: 47.4% vs. 71.6% in controls, *P* < 0.000 1; *Piezo1* at P14: 44.8% vs. 67.9% in controls, *P* < 0.000 1; *Trpv4* at P14: 45.3% vs. 63.7% in controls, *P* < 0.001) (Fig. S13c).

## Discussion

Despite significant progress in identifying skeletal stem cells (or progenitors) in bone and articular cartilage,^23,33^ the developmental trajectory of the tendon attachment unit requires deeper understanding. Previous research reported that the embryonic enthesis differentiates from in situ *Scx*^+^*Sox9*^+^ progenitors,^34^ which exhibit bilinear fate determination towards cartilage or tendon under the influence of shared regulatory elements.^11^ However, the specificity of these markers for embryonic enthesis progenitors is limited: *Scx* is widely expressed in tendon soft tissues, and *Sox9* is broadly expressed in chondrocytes during early limb development,^34–36^ meaning *Scx*^+^*Sox9*^+^ co-expression is not exclusive to the enthesis fibrocartilage layer.^24^ In contrast, the *Tnn*^+^ enthesis progenitors identified in this study are located exclusively at entheseal sites from embryonic through postnatal stages, addressing a gap in understanding how enthesis fibrochondrocytes are fate-determined in the embryo.

Literature review indicates that the extracellular matrix protein tenascin-W, also known as tenascin-N (TNN), was initially described in relation to osteogenesis and is expressed in mesenchyme and bone formation.^37^ TNN deficiency led to defects in the alveolar bone and periodontal ligament of the growing rodent incisors.^38^ Intriguingly, *Tnn* is also expressed in newly identified antler bud stem cells, which can differentiate into bone and cartilage following xenotransplantation.^39^ Notably, following in vivo implantation, these fluorescently labeled *Tnn*^+^ cells were no longer detected in newly formed cartilage, suggesting that *Tnn* expression is lost upon their differentiation into downstream lineages.^39^ Our spatial transcriptomic and lineage tracing results showed that a unique population of *Tnn*^+^ cells appeared in the enthesis area as early as embryonic day 14 (E14), distinct from tenocytes and epiphyseal chondrocytes. And the number of *Tnn*^+^ cells in the enthesis increased significantly between P0 and P7, well before the initiation of fibrocartilage differentiation, then gradually decreased with age, becoming scarcely detectable by 4 weeks postnatally. Similar in our scRNA-seq results, the cell heterogeneity in postnatal enthesis development (from P1 to P28) is not entirely similar, as we observe a notable decrease in the proportion of Quiescent progenitors between P1 and P7. This decrease coincides with the establishment of the enthesis progenitor and chondrocytes population. While the period after this (P14 to P28) shows more stability, suggesting that once the main “wave” of differentiation has occurred to form the fibrocartilage, the tissue enters a phase of maturation and homeostasis. In together with our pseudotime trajectory analysis and cell differentiation analysis results to find *Tnn* as driving gene in fibrocartilage differentiation, we propose that the *Tnn*^+^ population represents the specific subset of progenitors that are actively undergoing this differentiation into mature enthesis fibrochondrocytes.

It has been well established that the postnatal maturation of the enthesis is critically dependent on progenitor cells that generate its specialized fibrochondrocytes and extracellular matrix,^40^ seminal work by Thomopoulos et al. have identified Gli1 lineage cells as a key pool of resident enthesis progenitors and ultimately populate the entire mature enthesis.^13,24,41,42^ The *Tnn*^+^ progenitors identified in this study showed similarities with *Gli1*^+^ lineage in enthesis development, as a key similarity is that their early appearance during embryonic development and their shared destiny in forming the enthesis fibrocartilage, separate from the main lineages of tendon and cartilage cells. This study has confirmed that *Tnn*^+^ cells appear around embryonic day 14 (E14) and drive fibrocartilage differentiation, while the lineage tracing results reported by AG Schwartz et al. show that *Gli1*^+^ progenitor cells are also present at E14.5. This suggests both populations are fundamental to establishing the enthesis from its earliest stages and acting as specialized progenitors. However, despite the similarities between *Gli1*^+^ and *Tnn*^+^ cells in shared origin, a crucial distinction emerges in their postnatal dynamics and long-term roles. The Gli1^+^ population is maintained and expands after birth, persisting into adulthood as a stable, resident progenitor pool essential for continued growth, maturation, and homeostasis.^10,41,42^ This life-long presence makes *Gli1*^+^ lineage an ideal model for understanding the pathological processes of enthesis degeneration and injury-repair diseases. In stark contrast, the *Tnn*^+^ cell population in tendon enthesis is transient; it undergoes a rapid expansion in the first postnatal week (P1-P7) before becoming scarce by four weeks in mice. This transient profile suggests the *Tnn*^+^ population represents not a permanent reservoir, but instead a dynamic wave of progenitors which actively undergoing differentiation into mature chondrocytes right during the initiation of fibrocartilage formation (P7). This precisely defined temporal window makes the *Tnn*^+^ lineage an exceptional marker for dissecting the specific, coordinated events of enthesis construction during its most active phase of development.

In this study, *Tnn*^+^ cells were ubiquitously observed at the attachment sites of the rotator cuff, cruciate ligaments, patellar tendon, and Achilles tendon. Unlike the previous *Tnn*^+^ cells found in other ossification tissues, entheseal *Tnn*^+^ cells were not detected in the subchondral bone layer of tendon enthesis and did not express the osteogenic marker OSTERIX. Instead, a considerable portion of their progeny continued to express the mineralization-associated protein ALPL. This aligns with gene functional analysis results, which showed that entheseal *Tnn*^+^ cells are highly associated with processes such as chondrocyte differentiation, extracellular matrix synthesis, and proteoglycan synthesis. Crucially, in vivo ablation of *Tnn*^+^ cells postnatally resulted in an entheseal hypoplasia phenotype, characterized by a reduced number of fibrocartilage cells, compromised mechanical properties, and diminished mineralization. Altogether, these results demonstrate that *Tnn*^+^ enthesis progenitors are indispensable for the development and maturation of fibrocartilage at the tendon entheses.

This study confirmed the hypoplasia of tendon enthesis after unloading, as evidenced by reduced fibrocartilage matrix content and type II collagen, as well as reduced cell size fibrochondrocytes. These observations are consistent with the findings by Thomopoulos et al., who reported compromised fibrocartilage formation in unloaded entheses between 3 and 8 weeks postnatally,^16^ suggestive of the modulation of the number and function of enthesis progenitors by the different loading environment.^15^ In the current study, we analyzed the proportion of cells expressing widely known chondrogenic and osteogenic progenitor markers. In comparison with *Gli1*, *Pdgfra*, *Prrx1*, *Lepr*, *Ctsk*, *Ly6a*, *CD34*, and *Thy1*, the percentage of *Tnn*^+^ cluster was most significantly decreased in the unloaded group, which in together with the lineage tracing results in unloaded tendon entheses, indicating that *Tnn*^+^ cells represent a distinct population of enthesis progenitors that are responsive to mechanical stimuli. Another key finding of this study is the observed decrease in the expression of mechanosensitive ion channels *Piezo1* and *Trpv4*, and integrins *Itga5* and *Itgav*, within *Tnn*^+^ enthesis progenitors following enthesis unloading. Literature indicates that PIEZO1 is a well-established mechanosensitive ion channel that transduces mechanical stimulation (particularly tensile force) and enhances its permeability to cations, especially Ca²⁺.^43^ In chondrocytes, PIEZO1 is widely accepted to sense mechanical loads in joints and facilitate adaptation to these loads by regulating chondrocyte signaling and metabolic activities, thereby contributing to cartilage homeostasis and repair.^29,43^ Transient receptor potential vanilloid 4 (TRPV4) is also a non-selective cation channel with good selective permeability to calcium ions.^44^ TRPV4-mediated Ca^2+^ signaling was proven to play a central role in the transduction of mechanical signals to support cartilage extracellular matrix maintenance.^30,44^ The integrin family proteins connect the extracellular matrix (ECM) to the cytoskeleton and nucleus, and act as intermediaries for the transduction of mechanical signals.^31^ While our findings implicate these classic mechanosensitive molecules in *Tnn*^+^ cells, we acknowledge that precisely quantifying the reduction in mechanical stimuli at the single-cell level following BTX-induced muscle paralysis is challenging. Therefore, further experiments are required to draw definitive conclusions. Future research could involve isolating *Tnn*^+^ enthesis progenitors and applying controlled mechanical stimulation to these cells in vitro. Such an approach could elucidate the specific intracellular mechanisms by which *Tnn*^+^ cells sense and respond to mechanical stimuli.

Despite the promising findings, our study still has some limitations. In this study, we used diphtheria toxin into shoulder joint to ablate the *Tnn*^+^ cells in tendon entheses, and we found *Tnn*^+^ cells in the perichondrium/periosteum were also unavoidably ablated. This is an undeniable technical limitation of the local injection of DT. We observed a decrease in the quantity of *Tnn*^+^ enthesis progenitors following the removal of muscle loading. We also found that *Tnn* gene expression was downregulated in unloaded entheses, suggesting that *Tnn* expression in enthesis progenitors is mechanosensitive. However, the current study did not investigate the specific molecular mechanisms by which mechanical loading influences *Tnn* gene expression. Further in vitro experiments will be necessary in future research to address these important questions.

In summary, this study addressed the long-standing question of the cellular origin of enthesis fibrocartilage by constructing single-cell atlases of both normally developed and mechanically unloaded tendon entheses. We identified a novel population of *Tnn*^+^ enthesis progenitors and demonstrated that their quantity and function are significantly influenced by tendon unloading. These findings provide, at least in part, an answer to how mechanical loading regulates enthesis development. And the identification of additional genetic markers for enthesis progenitor lineages would provide an exceptional marker for dissecting the specific, coordinated events of enthesis construction during its most active phase of development.

## Materials and methods

### Mice

Tnn-Cre^ERT2^ mice were generated via CRISPR/Cas9 (Shanghai Model Organisms) by inserting 2A-CreERT2 into the into the ATG of *Tnn* gene; correct integration was verified by sequencing (supplementary files 2-4). These were crossed with Ai9 reporters for lineage tracing or R26-iDTR mice for cell ablation. For lineage tracing, tamoxifen was administered to pups (50 mg/kg) or pregnant dams (75 mg/kg), with specific timepoints indicated in figure legends. Model fidelity was validated via TNN immunofluorescence (Fig. S10a). For ablation, Tnn-Cre^ERT2^; R26-iDTR mice received tamoxifen (P1–P5) followed by repeated local Diphtheria Toxin (DT) injections (5 ng/g) starting at P3. All animal experimental protocols were approved by the Animal Ethics Committee of Central South University (No. 2022020058). Detailed genotyping, sequencing, and injection protocols are in the supplementary materials.

### Supraspinatus tendon enthesis unloading

A chronic enthesis unloading model was established in mice through repeated Botulinum toxin A (BtxA, Lanzhou Institute of Biological Products) injections.^15^ Specifically, 0.2 U of BtxA dissolved in 2 μL of saline solution was injected twice per week in the left supraspinatus muscle from birth until 4 weeks and then once per week through sacrifice at P56. Injections were performed using a 32-gauge syringe (Hamilton) to deliver a low volume of 2 µL to minimize diffusion of the toxin. The contralateral supraspinatus muscles were injected with the same amount of saline and served as controls. The effected of BtxA injection was confirmed by gross observe of supraspinatus muscle atrophy and weight change (Fig. S11).

### Cell isolation from the supraspinatus tendon enthesis

Supraspinatus entheses (*n* = 10–15/group) were micro-dissected from pooled sibling limbs. Samples were minced in chilled DMEM and digested with 1 mg/mL collagenase II, 20 ng/mL DNase, and 3% FBS at 37 °C for 60 min. The suspension was filtered (40 μm) and centrifuged at 500 g (4 °C) to pellet cells.

### 10X visium spatial sequencing and data analysis

Spatial sequencing was performed using the 10X Visium HD platform (v1 chemistry). Frozen, OCT-embedded sections were fixed in methanol, stained, and permeabilized for optimal times. After brightfield imaging, libraries were prepared and sequenced on an Illumina NovaSeq 6000. Data was processed using Space Ranger v.3.0.0 aligned to the mm10-2020-A reference genome. Further analysis details are in the supplementary materials.

### Single-cell RNA sequencing (scRNA-seq) and data analysis

scRNA-seq was performed on the MGI DNBelab C Series platform. Approximately 8 000–10 000 cells per group were encapsulated using the C4 scRNA Chip and processed with the DNBelab C Series Single-Cell Library Prep Set. Following quality control (Qubit/Bioanalyzer), libraries were sequenced on a DIPSEQ T1 platform. Raw reads were filtered, demultiplexed, and aligned to the mouse GRCm38 genome using the DNBC4 pipeline. Further analysis details are in the supplementary materials.

### Antibody staining and flow cytometry

Freshly isolated cells were resuspended into FACS buffer (PBS supplemented with 2% FBS) and stained with antibodies to remove blood and myeloid cells. DAPI (BD) staining was used to exclude dead cells. Flow cytometry was performed on BD FACS Aria II; all resultant cells were gated using doublet-discrimination parameters and collected into polypropylene FACS tubes.

### Cell culture and colony forming assay

FACS-purified BMSCs or PSCs were cultured at a density of 10 cell per cm^2^ in nutrient medium (αMEM, Corning) supplemented with 10% qualified FBS (Gibco), and 1% penicillin/streptomycin (ThermoFisher). The cultures were incubated at 37 °C in a gas-tight chamber (ThermoFisher) with 5% O_2_ and 10% CO_2_ for 7 days. The fibroblastic colonies were stained with Giemsa (1:20 in distilled water, Solarbio) and then counted under an AX10 microscope (Zeiss).

### Histological and immunochemistry of paraffin sections

Shoulder samples (P14, P28, P56) were fixed in 10% formalin, dehydrated in graded alcohols, and embedded in paraffin. 5 μm sections were stained with Hematoxylin/Eosin (H&E) or Toluidine blue/Fast green. Renal capsule transplants were stained with H&E. All sections were imaged using an Olympus light microscope.

### Immunofluorescence staining of frozen sections

OCT (Thermo Scientific) embedded enthesis samples were sectioned into 10 µm ice-sections, using the CryoJane tape-transfer system (Leica Biosystems, 39475208 and 39475214). For immunofluorescence staining, the sections were washed with PBS, permeabilized with 0.1% TritonX-100, and blocked with 3% bovine serum albumin (BSA; Sigma-Aldrich-Aldrich). Then incubated with primary antibodies at 4 °C overnight, then incubated with secondary antibody at room temperature for 1 h and counterstained with DAPI (Invitrogen, USA). All the images were observed and captured using a Zeiss AxioImager.M2 microscope (Zeiss) equipped with an Apotome2 System. Positive cells of each captured image were measured using 200x magnification graphs for each slide by the Image J software (National Institutes of Health, Bethesda). The antibodies used in this study are listed in Table S1.

To evaluate the distribution of *Tnn*^+^ cells, the 10 μm thick cryosections from TnnCre^ERT2^; tdTomato mice were washed with PBS and stained with DAPI, then directly captured using a Zeiss Apotome system. To evaluate the differentiation of *Tnn*^+^ cells, the cryosections were washed in 0.1% Triton X-100/PBS, blocked in 3% bovine serum albumin, and incubated with primary antibodies at 4 °C overnight and appropriate secondary antibodies for 1 h at room temperature.

### Synchrotron radiation micro-computed tomography (SR-μCT)

SR-μCT was performed at the BL13W1 beamline of the Shanghai Synchrotron Radiation Facility. Formalin-fixed samples were scanned at 18.0 keV with effective voxel sizes of 0.65 µm or 3.25 µm (Fig. S12). Images were reconstructed using PITRE software. Bone morphological parameters (BV/TV, Tb.Th, Tb.N) were quantified using CT Analyzer v1.11, and 3D visualization was generated with Amira v2022. Cell lacunae within the fibrocartilage were segmented and analyzed for volume, density, and sphericity. Detailed scanning, reconstruction, and segmentation protocols are available in the supplementary information.

### Tendon enthesis biomechanical test

Biomechanical testing was performed on supraspinatus tendon-humerus samples using an Instron 5942 system. The humerus was fixed and the tendon freeze-clamped 1–2 mm from the insertion. Samples were preconditioned and loaded to failure at 0.03 mm/s. The insertion footprint area was calculated from μCT-based 3D reconstructions (Amira) to normalize data. Material properties (force, stress, modulus, stiffness) were derived from force-displacement curves. Detailed protocols and exclusion criteria are provided in the supplementary information.

### Transmission electron microscopy

Samples were fixed in glutaraldehyde, stained with osmium tetroxide, and embedded in Spurr resin. 100 nm sections were prepared using a Leica EM UC7 ultramicrotome. Initial imaging and EDX were performed on an FEI Tecnai G2 F20 at 200 kV. Advanced analysis (HAADF-STEM, STEM-EDX, SAED, and STEM-EELS) was conducted on an aberration-corrected FEI Titan G2 80-200. Ca/P ratios and EELS spectra were analyzed to assess mineral composition and structural details. Detailed preparation and calibration protocols are available in the supplementary information.

### Quantitative real-time PCR

Supraspinatus tendon entheses (from normal vs unloaded groups or *Tnn*^-/-^ vs *Tnn*^+^ cell ablation groups) at day 14, 28, and 56 were dissected, liquid nitrogen frozen, and pulverized in a ball mill homogenizer (Servicebio, China). Total RNA was purified using the RNeasy Kit (Qiagen, Germany). Reversed transcription was performed with High-Capacity cDNA Reverse Transcription Kit (Applied Biosystems, USA), according to the manufacturer’s protocol. Quantitative real-time PCR was performed using Fast SYBR Green master mix (Applied Biosystems) on the Applied Biosystems QuantStudio 7 Flex System. Glyceraldehyde 3-phosphate dehydrogenase (GAPDH) serves as housekeeping gene, and the relative mRNA expression level for each target gene was determined as 2^-ΔΔCt^. Primer sequences are given in Table S2.

### Statistical analysis

Statistical analyses were performed using GraphPad Prism 9. The Shapiro-Wilk test was used to assess the normality of data distribution. For comparisons between two groups, a two-tailed Student’s *t*-test was used for normally distributed data, while the Mann-Whitney U test was used for non-normally distributed data. For comparisons among three or more groups, two-way ANOVA followed by Tukey’s post hoc test was employed. A *P*-value < 0.05 was considered statistically significant. Detailed statistical methods are specified in the figure legends.

## Supplementary information

supplementary materials

Supplementary file 1

Supplementary file 2 Tnn-2A-CreERT2 Donor Vector sequence.dna

Supplementary file 3 Tnn-2A-CreERT2 Recombinated Genomic DNA sequence.dna

Supplementary file 4 Tnn-2A-CreERT2 Wild Type Genomic DNA sequence.dna

## Data Availability

Single-cell sequencing raw datasets created in this study are publicly available at NCBI BioProject (PRJNA1336302) database. Spatial transcriptomic h5ad file is available on GitHub (https://github.com/zt6597/enthesis-development). Any additional information required to re-analyze the data in the paper is available from the corresponding author upon request.
