# Supplementary material for: Identification of a new population of Tnn+ progenitors to form tendon enthesis fibrocartilage
Source: Bone Res. 2026 Apr 21;14:43. doi: 10.1038/s41413-026-00519-3 (PMC13096267; doi:10.1038/s41413-026-00519-3)
Supplement: Supplementary file 1 — supplementary materials [file 41413_2026_519_MOESM1_ESM.docx]

**Identification of a new population of Tnn^+^ progenitors to form tendon enthesis fibrocartilage**

**Supplementary information**

**Supplementary materials and methods**

**Supplementary Table 1.** Antibodies used in this study.

**Supplementary Table 2.** Primer sequences used in this study.

**Figure S1.** Spatially resolved transcriptomic profiling of mouse embryonic tendon enthesis.

**Figure S2.** scRNA-seq data processing, quality control, and cluster annotation.

**Figure S3.** Cell fate determination and chondrogenic lineage dynamics within enthesis subclusters.

**Figure S4.** Similarities and differences between Gli1^+^ and Tnn^+^ cells in enthesis chondrogenesis.

**Figure S5.** Two spatially and phenotypically distinct populations of Tnn-tdTomato⁺ cells.

**Figure S6.** In vivo distribution of and in vitro potency of Tnn^+^ cells.

**Figure S7.** Ablating the Tnn+ enthesis progenitors inhibits fibrocartilage development.

**Figure S8.** Enthesis chondrogenesis is hampered after tendon unloading.

**Figure S9.** Comparison of the number of Tnn+ cells with other classical stem cell subpopulations in enthesis chondrogenic trajectory.

**Figure S10.** Validation of Tnn reporter with immunohistochemistry and the efficiency of Tnn ablation model.

**Figure S11.** Validation of BTX-induced supraspinatus muscle atrophy.

**Figure S12.** Schematic diagram of the stabilization setup and enthesis surface definition in SR-μCT experiments.

**Figure S13.** Expressions of previous reported mechanosensitive channels in Tnn^+^ cells were significantly decreased after the loss of mechanical stimulation.

**Supplementary materials and methods**

Mice

Wildtype C57BL/6 mice were purchased from Hunan SJA Laboratory Animal Company (China). Tnn-Cre^ERT2^ mice was generated by homologous recombination using CRISPR/Cas9 technology. A cDNA encoding Cre (2A Peptide-CreERT2) was inserted into the ATG of the *Tnn* gene before 3’ UTR, utilizing the guide RNA (gRNA) 5'-AGCGCTCCATAGGAAAAGCA-3'. Positive F0 founder mice were crossed with wild-type C57BL/6J mice to generate the F1 generation. F1 offspring were genotyped by PCR, and the correct integration was verified by sequencing (see supplementary files for sequencing data). Tnn-Cre^ERT2^ mice were generated by Shanghai Model Organisms Co., Ltd.

R26^CAG-LSL-tdTomato-2A-DTR^ mice (stock number: NM-KI-210094) were obtained from Shanghai Model Organisms Co., Ltd. Ai9 strain (R26^Sortm9(CAG-tdTomato)Hze/J^, Rosa26-tdTomato) was purchased from Jackson Laboratory (USA). All the mice were genotyped according to the recommended PCR-based protocol.

*Tnn*^+^ lineage tracing (Tnn-Cre^ERT2^; Rosa26-tdTomato) mice were generated by crossing Tnn-Cre^ERT2^ mice with R26^Sortm9(CAG-tdTomato)Hze/J^ mice. To perform pulse-chase lineage tracing, Cre was induced via a short-term tamoxifen intraperitoneal (IP) injection. For infant induction, pups received five consecutive daily injections of tamoxifen (50 mg/kg body weight). For fetal induction, pregnant dams received daily injections of tamoxifen (75 mg/kg body weight). The specific start day for each treatment (embryonic or postnatal day) is indicated in the corresponding figure legends. Following this induction "pulse," the mice were "chased" for various periods, and tissues were harvested at later time points (P7, P14, P28, P56) without any further tamoxifen treatment. To validate that our *Tnn*^+^ lineage tracing mice accurately marks TNN-expressing cells, we performed co-immunofluorescence for the TNN protein. We observed strong co-localization between the tdTomato reporter and TNN protein staining (Fig. S10a), confirming the fidelity of our genetic model in identifying the correct cell population.

To ablate *Tnn*^+^ cells, Tnn-Cre^ERT2^ mice were crossed with R26^CAG-LSL-tdTomato-2A-DTR^ mice. Cre recombination was induced starting at postnatal day 1 with five consecutive daily intraperitoneal injections of tamoxifen (50 mg/kg body weight). When two days after the first tamoxifen injection (at P3), mice received 3 days of first phase daily injection of Diphtheria Toxin (DT; 5ng/g body weight, Sigma-Aldrich) into the right shoulder, followed by one time DT injection every 3 days until the age of 4 weeks to ensure continued ablation as new cells might be differentiating. Tnn-Cre^ERT2^; tdTomato-iDTR mice without DT injection served as genetic controls.

Male mice were used for *Tnn*^+^ cell ablation and tendon unloading experiments. Mice of both sexes were used for other experiments. All mice were kept in the specific pathogen-free facility of the Laboratory Animal Research Center of Central South University at 22-24°C with a 12 h dark/light cycle, and with regular diet. All animal experimental protocols were approved by the Animal Ethics Committee of Central South University (No. 2022020058).

10X Visium Spatial Sequencing and data generation

The spatial sequencing experiments were carried on 10X visium hd platforms. Frozen samples were embedded in OCT (TissueTek Sakura) and cryosectioned at -10 °C (Thermo Cryostar). Sections were placed on chilled Visium Tissue Optimization Slides and Visium Spatial Gene Expression Slides. Tissue sections were then fixed in chilled methanol and stained according to the Visium Spatial Gene Expression User Guide. For gene expression samples, tissue was permeabilized for optimal time based on tissue optimization time-course experiments, with chemistry Visium HD v1 kit. Brightfield histology images were taken and raw images were stitched together as .tiff files with low- and high-resolution settings. Libraries were prepared and loaded on a NovaSeq 6000 System (Illumina. The raw FASTQ files and histology images were processed by using the Space Ranger software v.3.0.0, against the Cell Ranger mm10 reference genome refdata-cellranger-mm10-2020-A (<https://cf.10xgenomics.com/supp/cell-exp/refdata-gex-mm10-2020-A.tar.gz>).

Histology image processing and segmentation

Histology images were processed and nuclei were segmented by using the StarDist H&E model in Python, with prob_thresh=0.001, nms_thresh=0.001, based on the steps reported by 10X genomics (https://www.10xgenomics.com/analysis-guides/segmentation-visium-hd).

Spatial clustering and gene module identification

Spatial clustering was performed by BASS in R (https://zhengli09.github.io/BASS-Analysis/index.html), which utilizes a Bayesian hierarchical modeling framework to perform clustering analysis. The BASS codes were followed by default document, with C = 21, R = 21, and all the spatial clusters were annotated manually based on its anatomical location.

Interpretable spatially aware dimension reduction was performed by using the time series training model provided by STAMP in python (https://jinmiaochenlab.github.io/scTM/notebooks/stamp), according to its official document, with n_topics fit to 6. The top 100 highest relative genes of each gene topic were used as input to perform GO ontology annotation by using GSEApy in python.

Single-cell RNA sequencing (scRNA-seq) and library construction

The scRNA-seq experiments were carried DNBelab C Series platform (MGI) platforms. 8000-10,000 cells were loaded for each group by using the DNBelab C Series Single-Cell Library Prep Set (MGI, 1000021082) according to the manufacturer’s instructions. Cells were loaded on a C4 scRNA Chip to encapsulate single cell droplets. After 20 minutes of mRNA hybridization with capture beads at room temperature, droplets were damaged to collect beads. Reverse transcription (RT) was performed and collected for second-strand cDNA synthesis. The cDNA and Oligo products were used to generate standard barcoded sequencing libraries. Libraries were quantified using Qubit 3.0 with a Qubit TM dsDNA HS Assay Kit (Thermo Fisher Scientific, Q10212) and quality was assessed via Agilent Bioanalyzer. The resulting products were further subjected to sequencing library construction and sequenced in a DIPSEQ T1 platform. The DNBC4 pipeline (https://github.com/MGI-tech-bioinformatics/DNBelab_C_Series_HT_scRNA-analysis-software) was used to filter, demultiplex, and align the raw reads to the mouse reference genome GRCm38.

Quality control, scale and integration of sc-Seq datasets

All the sample matrices were loaded by the Seurat package (v4.3.0, https://satijalab.org/seurat/), cells with poor quality were removed (less than 5th percentile genes, or more than 10% mitochondrial genes). Doublets were detected and removed by using R package Doubletfinder^47^. And we used computational tools (SoupX) to detect and remove technical artifacts like doublets, multiplets, and ambient RNA to minimize the effect of contamination^48^. After quality control, all the filtered datasets were scaled with the Sctransform (V2) algorithm to avoid unwanted variation including excessive percentages of mitochondrial reads and cell cycle effect. Then the datasets were batch-corrected by using Seurat FindIntegrationAnchors() with default parameters. Finally, the integrated datasets were subclustered to exclude uninterested clusters (including articular chondrocytes, osteocytes, immune cells, red blood cells, endothelial cells, smooth muscle cells, and proliferative cells).

Dimensionality Reduction, Clustering, and DEGs analysis

Uniform Manifold Approximation and Projection (UMAP) was used to visualize the dataset in low dimensions. K-nearest neighbor (KNN) method and the Louvain algorithm were applied to cluster the cells, with 50 PCs selected. The FindAllMarkers function in Seurat was used to calculate differentially expressed genes (DEGs) among different cell clusters, with the ‘test.use’ function set to a statistical framework called MAST. Genes met the criteria that 1) expressing in a minimum fraction of 10% in either of the two tested populations; 2) at least a 0.1-fold difference (log-scale) between the two tested populations; 3) adjusted P values less than 0.01, were considered as signature genes. Clusters were annotated according to the expression of those highly variable genes reported in the literature.

Cell trajectory analysis

Python package Cellrank with adapted CytoTRACE kernel implementation was used (https://github.com/theislab/cellrank) to compute the terminal cell state and cluster absorption probabilities. We also fit Generalized Additive Models (GAMs) onto the fibrocartilage trajectory to weight each cells contribution according to its vector of fate probabilities, and to plot the trajectory-specific gene expression trends. The most driving genes in fibrocartilage trajectory were calculated by using compute_lineage_drivers method. The pseudotime analysis of chondrogenic and tenogenic lineage was conducted by using Slingshot (https://github.com/kstreet13/slingshot) with default parameters, the start of lineage was set to mesenchymal progenitors, and end clusters was set to enthesis chondrocytes and tenocytes. The

Differential abundance testing with Milo

Differential cell-state abundances of enthesis related clusters between BTX and normal groups were calculated by using the MiloR package (https://github.com/MarioniLab/miloR). Specifically, a K-nearest neighbors (KNN) graph was built using the graph ‘PCA’ slot from the adjacency matrix of the processed Seurat object with the parameters: k = 10 and d=30. Cells were assigned to the neighborhoods based on the KNN graph using the ‘makeNhoods’ function (prop=0.1). To explore variations in cell counts between neighboring wound healing points (pairwise comparisons), cells from each group in each neighborhood were counted. Differential neighborhood abundance testing was performed using a generalized linear model (GLM). Differentially abundant cell neighborhoods with SpatialFDR ≤ 0.1 were plotted using the ‘plotNhoodGraphDA’ function.

Gene enrichment analysis

GO enrichment of cluster differentially expressed genes was performed by using the R package clusterProfiler (https://github.com/YuLab-SMU/clusterProfiler), with a Benjamini-Hochberg (BH) multiple testing adjustment and a false-discovery rate (FDR) cutoff of 0.1. The normalized gene expression matrix from Seurat DEGs were used as inputs. Module scores for each gene set were calculated using the AddModuleScore function implemented in Seurat. Gene sets used for scoring were selected from the Gene Ontology Browser of MGI Database (https://www.informatics.jax.org/vocab/gene_ontology).

Gene Trajectory Inference

Gene Trajectory Inference was performed by GeneTrajectory method (https://github.com/KlugerLab/GeneTrajectory). Briefly, the expression matrix of enthesis chondrocytes, enthesis progenitors and mesenchymal progenitors were used as input data. A cell-cell kNN graph in which each cell connected to its k-nearest (k = 20) neighbors. Pairwise graph-based Wasserstein distance was calculated between gene distributions, then a low-dimensional representation of genes (using Diffusion Map) based on the gene-gene Wasserstein distance matrix was mapped.

Synchrotron radiation micro-computed tomography (SR-μCT)

Three-dimensional morphology of tendon enthesis was evaluated by SR-μCT at the radiograph imaging and biomedical application beamline (BL13W1) of Shanghai Synchrotron Radiation Facility in China. SR-μCT has superior 3D visualization capacity of cartilage than conventional absorption imaging.

In brief, the supraspinatus-humerus samples (from normal vs unloaded groups or *Tnn*^-/-^ vs *Tnn*^+^ cell ablation groups) were dissected at day 56, and fixed in 4% formalin for 24 hours, then firmly wedged into a 200 µL pipette tip, with the bony portion of the sample secured within the main body of the tip. The pipette tip acted as a custom sample holder was securely mounted onto the scanner's rotating stage through a set screw and hot-melt adhesive (supplementary Fig. 12). During scanning, samples were placed at the center of the rotary stage and scanned with an angular step of 0.25º over an angular range of 180º. The beam energy and sample-to-detector distance were set to 18.0 keV and 10.0 cm respectively. A total of 900 radiographic projections were captured by the charge-coupled device detector (CCD). The high-resolution scans (10x lens) had a voxel size of 0.65 µm/pixel and the lower resolution scans (2x lens) for 3.25 µm/pixel. Dark- and flat-field images were captured to correct the electronic noise and variations in the X-ray source brightness. These projected radiographs were sequentially phase retrieved and transformed into 8-bit slices by PITRE software written by BL13W1.

Bone was extracted from soft tissue or bone marrow by threshold segmentation and a median filter was used to reduce noise. Morphological parameters of the newly formed bone were calculated with investigators blinded to groups, using data analysis software (CT Analyzer v1.11, Bruker Corporation, Germany), such as bone volume fraction (BV/TV), trabecular thickness (Tb.Th), and trabecular number (Tb.N). Consequent cross-sectional images of SST enthesis were used to perform 3D reconstruction and visualization of cartilage and subchondral bone by using 3D visualization software (Amira v2022, Thermo Scientific).

For cell quantification, cell lacunae were defined by hysteresis thresholding within fibrocartilage layer. For segmentation process, we used watershed algorithm and random-walk distance map to separate connected cells. All lacunae connected to the image borders were erased from the labeled volume. Each cell lacuna label was conducted with quantitative analysis including lacunar volume, lacunar surface area, density, as well as the sphericity of lacuna. Due to the cellular size and morphology between osteocyte and chondrocyte being quite different, we filter the chondrocyte by its volume and its location to subchondral bone.

Tendon enthesis biomechanical test

Supraspinatus tendon - humeral bone samples (from normal vs unloaded groups or *Tnn*^-/-^ vs *Tnn*^+^ cell ablation groups) were dissected at day 28 and 56 respectively. An Instron biomechanical testing system (model 5942, Instron, MA) was used to detect enthesis biomechanics. Specifically, the humeral head and the portion below it was rigidly fixed to prevent any motion during testing. The bone was oriented such that the tendon's line of action is aligned with the tensile axis of the testing machine. A freeze clamp was employed to freeze the supraspinatus tendon end into a solid block and securely gripped, and the freeze clamp was placed 1-2 mm to the bony insertion. The samples were tested at room temperature, and samples were preconditioned with 0.1 N and then loaded to failure at a rate of 0.03 mm/s. A consistent gauge length was used throughout the test. Data were excluded if the tendon slipped out of the grip or did not fail at the supraspinatus attachment site.

The supraspinatus-humerus μCT datasets and the 3D visualization software (Amira) were used to calculate the cross-sectional area which was defined as the SST insertion footprint area. The bony boundaries of the footprint area were determined from the cross-sectional images in the sagittal and coronal planes, which is typically demarcated by the bony transverse ridge between the articular surface of the humeral head and the greater tuberosity. The perimeter of the footprint boundaries on the humeral head was manually delineated on the 3D reconstructed volume of the greater tuberosity, then the surface area of this delineated ROI was automatically calculated. The force-displacement data were recorded to evaluate enthesis material properties (maximum force, stress, Young’s modulus, and stiffness).

Transmission electron microscopy

Supraspinatus tendon enthesis samples (from *Tnn*^-/-^ vs *Tnn*^+^ cell ablation groups) were dissected at day 56. The specimens underwent primary fixation in a solution of 2.5% glutaraldehyde in phosphate buffer (0.1M, pH 7.2) for a period of 24 hours, then rinsed with PBS and stained in 1% osmium tetroxide (OsO4) solution. Subsequently, the samples were dehydrated and embedded in a Spurr-based resin. Cross-sectional preparation of the samples was performed using an Lerca EM UC7 ultramicrotome, with sections cut to a thickness of 100 nm and placed onto 300-mesh nickel grids. Initial bright-field transmission electron microscopy (TEM) imaging and energy-dispersive X-ray (EDX) analysis of the osteochondral interface were conducted using a field-emission FEI Tecnai G2 F20 microscope at an acceleration voltage of 200 kV. Further advanced analyses, including high-angle annular dark-field scanning transmission electron microscopy (HAADF-STEM), STEM-EDX mapping, selected area electron diffraction (SAED), and scanning transmission electron microscopy with energy-dispersive X-ray spectroscopy (STEM-EELS), were executed on an aberration-corrected FEI Titan G2 80-200 microscope equipped with a Super-X EDX detector. The electron probe was calibrated with a DCOR plus spherical aberration corrector using a gold standard sample prior to each experiment. The calcium-to-phosphorus (Ca/P) ratios across various zones within the interface were meticulously calculated to assess the mineral composition. Additionally, EELS spectrum images were acquired from regions of interest within the energy range of 250 to 400 eV, focusing on the fine structural details of carbon (C K-edge) and calcium (Ca L2,3-edge).

**Supplementary tables**

**Supplementary Table 1.** Antibodies used in this study.

| **Antibody (clone)** | **Vendor** | **Dilution** | **Usage** |
| --- | --- | --- | --- |
| Alexa Fluor® 700 anti-mouse TER-119 (TER-119) | BioLegend | 0.25 ug/million cells | FACS |
| Alexa Fluor® 700 anti-mouse CD45 (I3/2.3) | BioLegend | 0.25 ug/million cells | FACS |
| Alexa Fluor® 700 anti-mouse LIN cocktail | BioLegend | 10 ul/million cells | FACS |
| APC anti-mouse LIN cocktail | BioLegend | 10 ul/million cells | FACS |
| DAPI (AB_2869624) | BD Pharmingen | 0.5 ul/test | FACS |
| Anti TNN (PA5-116020) | Thermo Fisher | 1:200 | IF |
| Anti SOX9 (ab185966) | Abcam | 1:200 | IF |
| Anti SCX (sc-518082) | Santa Cruz | 1:200 | IF |
| Anti ACAN (13880-1-AP) | Proteintech | 1:200 | IF |
| Anti SOST (21933-1-AP) | Proteintech | 1:200 | IF |
| Anti BSP (PA5-114915) | Thermo Fisher | 1:200 | IF |
| Anti ALPL (sc-398461) | Santa Cruz | 1:100 | IF |
| Anti OSTERIX (ab209484) | Abcam | 1:200 | IF |
| anti CTSK (11239-1-AP) | Proteintech | 1:200 | IF |
| Anti PIEZO1 (MA5-32876) | Thermo Fisher | 1:200 | IF |
| Anti TRPV4 (ab314454) | Abcam | 1:200 | IF |
| Anti MKI67 (ab15580) | Abcam | 1:200 | IF |
| Anti COL2A1 (28459-1-AP) | Proteintech | 1:300 | IHC |
| Anti COL1A1 (CL594-67288) | Proteintech | 1:400 | IHC |
| Anti-Rabbit IgG (Alexa Fluor® 488) (ab150073) | Abcam | 1:400 | IF |
| Anti-Rabbit IgG (Alexa Fluor® 594) (ab150064) | Abcam | 1:400 | IF |

**Supplementary Table 2.** Primer sequences used in this study.

| Genes | Forward (5’→3’) | Reverse (5’→3’) |
| --- | --- | --- |
| GAPDH | AGGTCGGTGTGAACGGATTTG | GCTTGACGTGTGGCTTGTTC |
| Sox9 | GAGCCGGATCTGAAGAGGGA | GCTTGACGTGTGGCTTGTTC |
| Acan | CCTGCTACTTCATCGACCCC | AGATGCTGTTGACTCGAACCT |
| Col1a1 | GCTCCTCTTAGGGGCCACT | CCACGTCTCACCATTGGGG |
| Col2a1 | GGGAATGTCCTCTGCGATGAC | GAAGGGGATCTCGGGGTTG |
| Col9a1 | CGACCGACCAGCACATCAA | AGGGGGACCCTTAATGCCT |
| Sost | AGCCTTCAGGAATGATGCCAC | CTTTGGCGTCATAGGGATGGT |
| Ibsp | ATGGAGACGGCGATAGTTCC | CTAGCTGTTACACCCGAGAGT |
| Alpl | CCAACTCTTTTGTGCCAGAGA | GGCTACATTGGTGTTGAGCTTTT |
| Mef2c | ATCCCGATGCAGACGATTCAG | AACAGCACACAATCTTTGCCT |
| Clec3a | ATGGCAAAGAACGGACTTGTC | CAACTTGAGATTTCAGGTCGTCA |
| Gli1 | CCAAGCCAACTTTATGTCAGGG | AGCCCGCTTCTTTGTTAATTTGA |
| Ptch1 | AAAGAACTGCGGCAAGTTTTTG | CTTCTCCTATCTTCTGACGGGT |

**Supplementary figures**


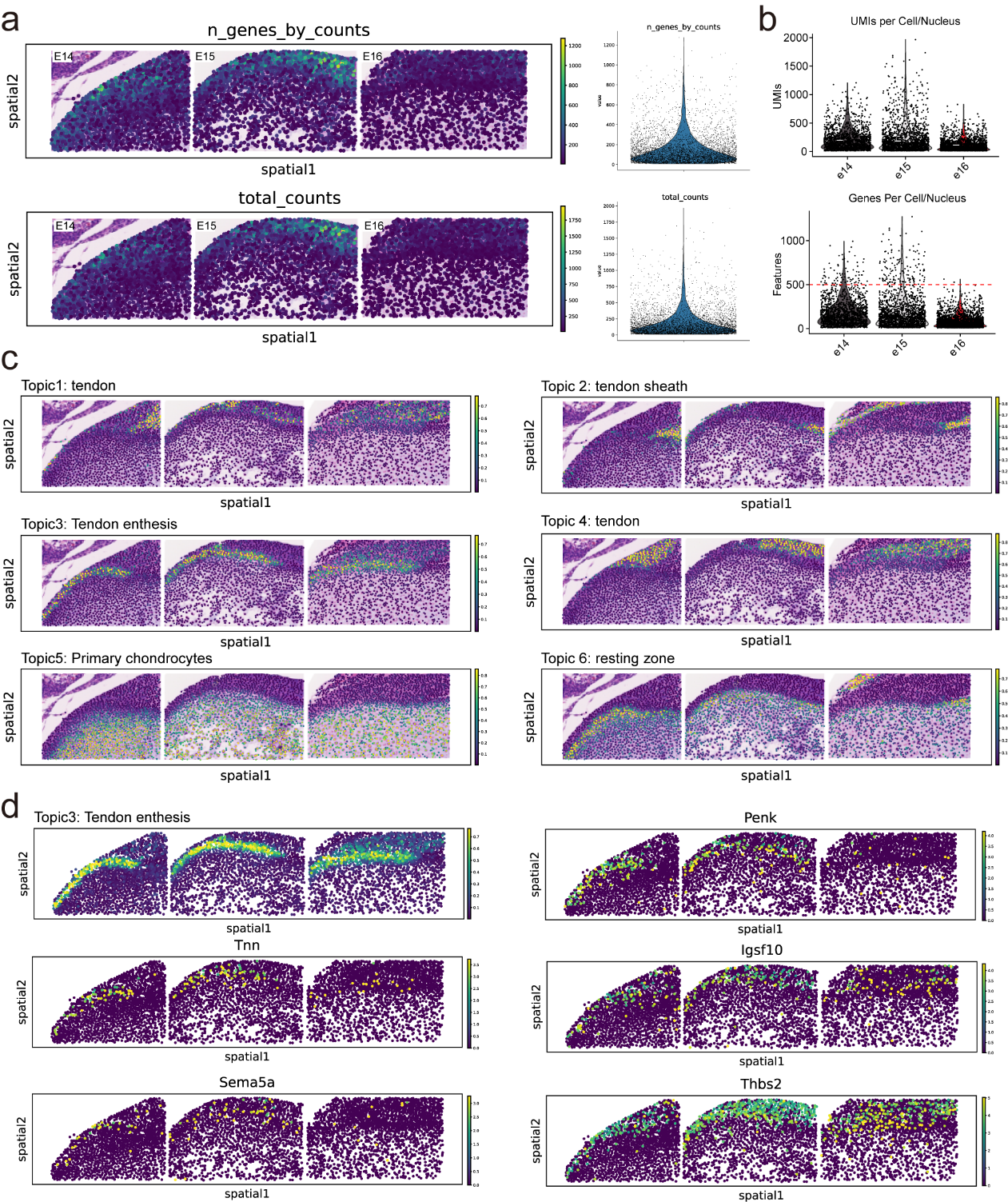


**Figure S1. Spatially resolved transcriptomic profiling of mouse embryonic tendon enthesis. a, b** Quality control (QC) metrics for Regions of Interest (ROIs) defined by nuclear segmentation within tendon enthesis tissue at embryonic day 14 (E14), E15, and E16. **c** Spatial Transcriptomics Analysis and Mapping Platform (STAMP) identifying distinct anatomical regions and cell zones, including tendon, tendon enthesis, tendon sheath, primary chondrocytes, and resting zone chondrocytes. **d** Spatial visualization of expression levels for the highest-ranking genes within the enthesis-specific gene module (gene topic 3) identified by STAMP algorithm.

**
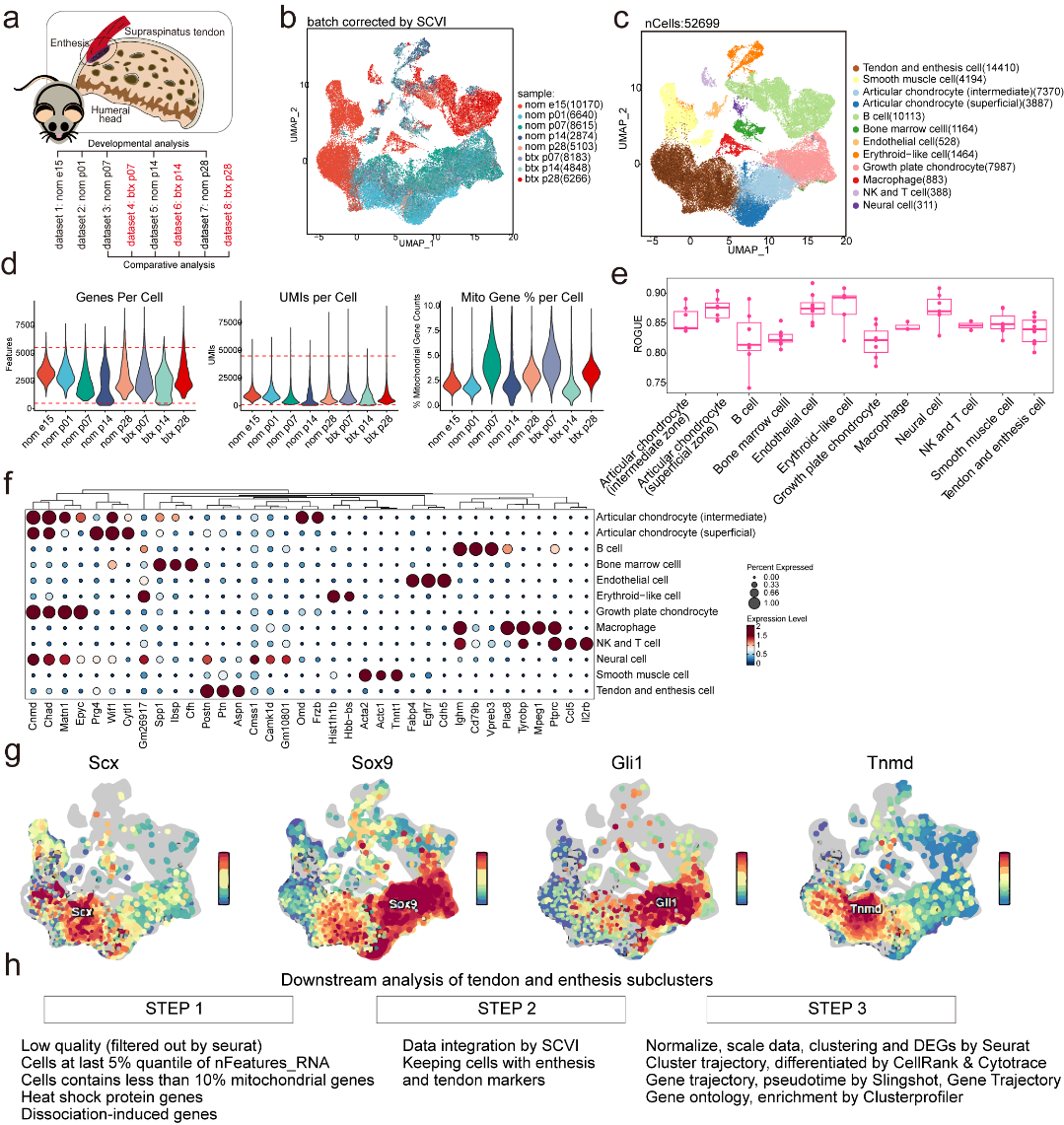
**

**Figure S2. scRNA-seq data processing, quality control, and cluster annotation. a** Schematic of the single-cell RNA sequencing (scRNA-seq) experimental design. **b, c** UMAP visualization of datasets following batch correction using SCVI, illustrating effective data integration. **d** Key quality control (QC) metrics for each scRNA-seq sample, including: (i) number of genes detected per cell, (ii) number of Unique Molecular Identifiers (UMIs) per cell, (iii) percentage of mitochondrial gene expression per cell, and (iv) cell complexity. **e** Assessment of cell cluster purity using ROGUE (Ratio of Global Unshifted Epsilon) scores. All identified clusters achieved high purity (ROGUE scores > 0.8). Boxplots display the median ROGUE score (center line), with lower and upper hinges representing the 25th and 75th percentiles, respectively. **f** Dot plot visualizing the expression of the top 3 marker genes for each identified cell cluster. **g** Feature plots illustrating the expression of representative enthesis-enriched genes across all cell clusters. **h** Overview of the scRNA-seq data analysis workflow, highlighting key software packages employed.


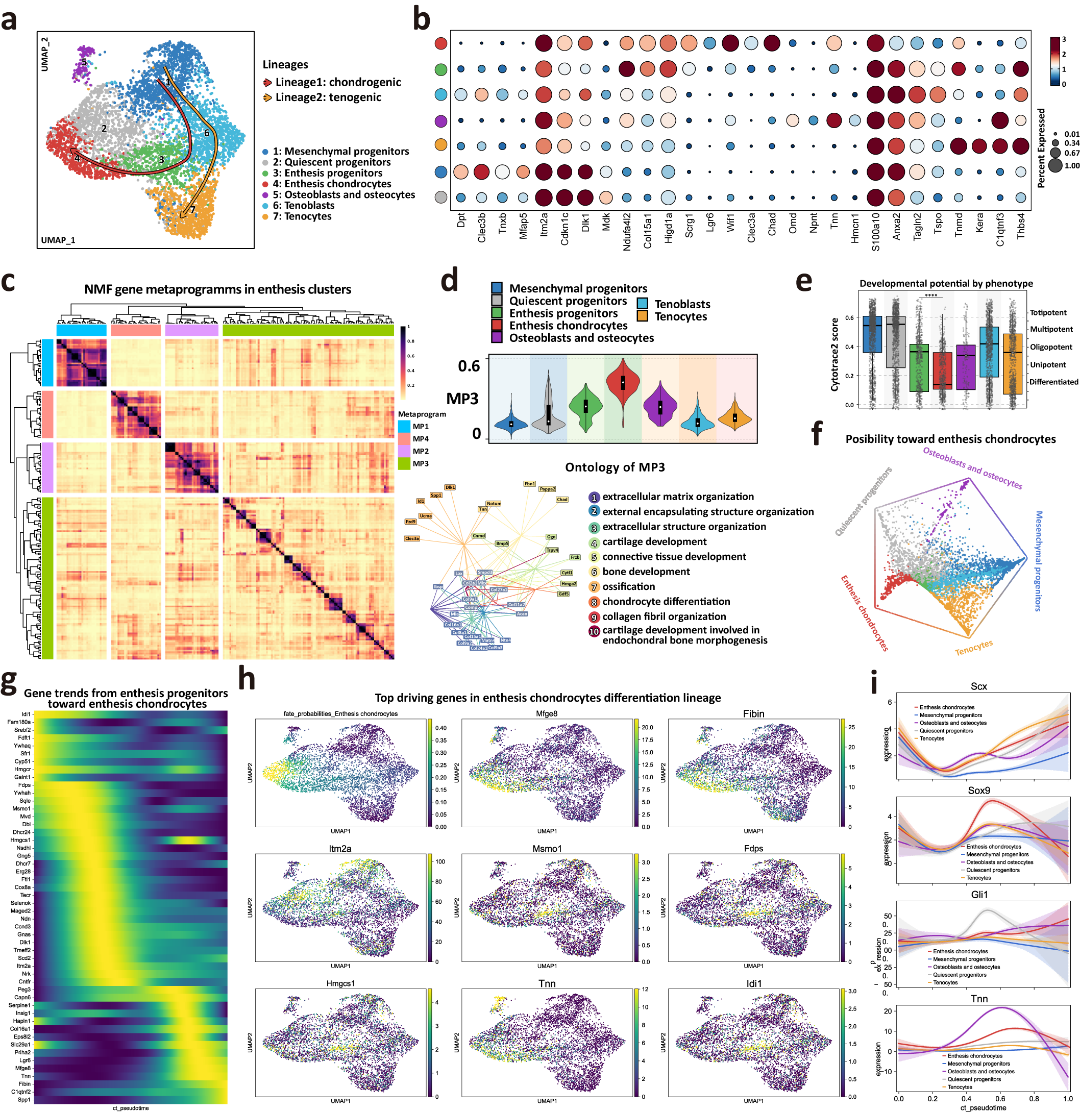


**Figure S3. Cell fate determination and chondrogenic lineage dynamics within enthesis subclusters. a** Uniform Manifold Approximation and Projection (UMAP) plot visualizing the inferred chondrogenic and tenogenic differentiation trajectories within enthesis-related cell clusters. **b** Dot plot displaying the expression of the top 3 marker genes for each identified cell subcluster. **c, d** Heatmap illustrating four major meta-programs (MPs) identified by Non-negative Matrix Factorization (NMF), with functional annotation (D) showing MP4 specifically enriched in enthesis chondrocytes and associated with ECM organization and cartilage development. **e** Assessment of developmental potency for each cell cluster, calculated using CytoTRACE. **f** Circular projection plot depicting the fate probabilities of each cell cluster towards the terminal enthesis chondrocyte state. **g** Heatmap visualizing dynamic gene expression changes along the inferred chondrogenic differentiation trajectory. **h** Feature plots highlighting the expression of top driving genes implicated in enthesis chondrocyte differentiation lineage. **i** Line plots showing the expression profiles of *Sox9*, *Scx*, *Gli1*, and *Tnn* along the different pseudotime trajectory

**
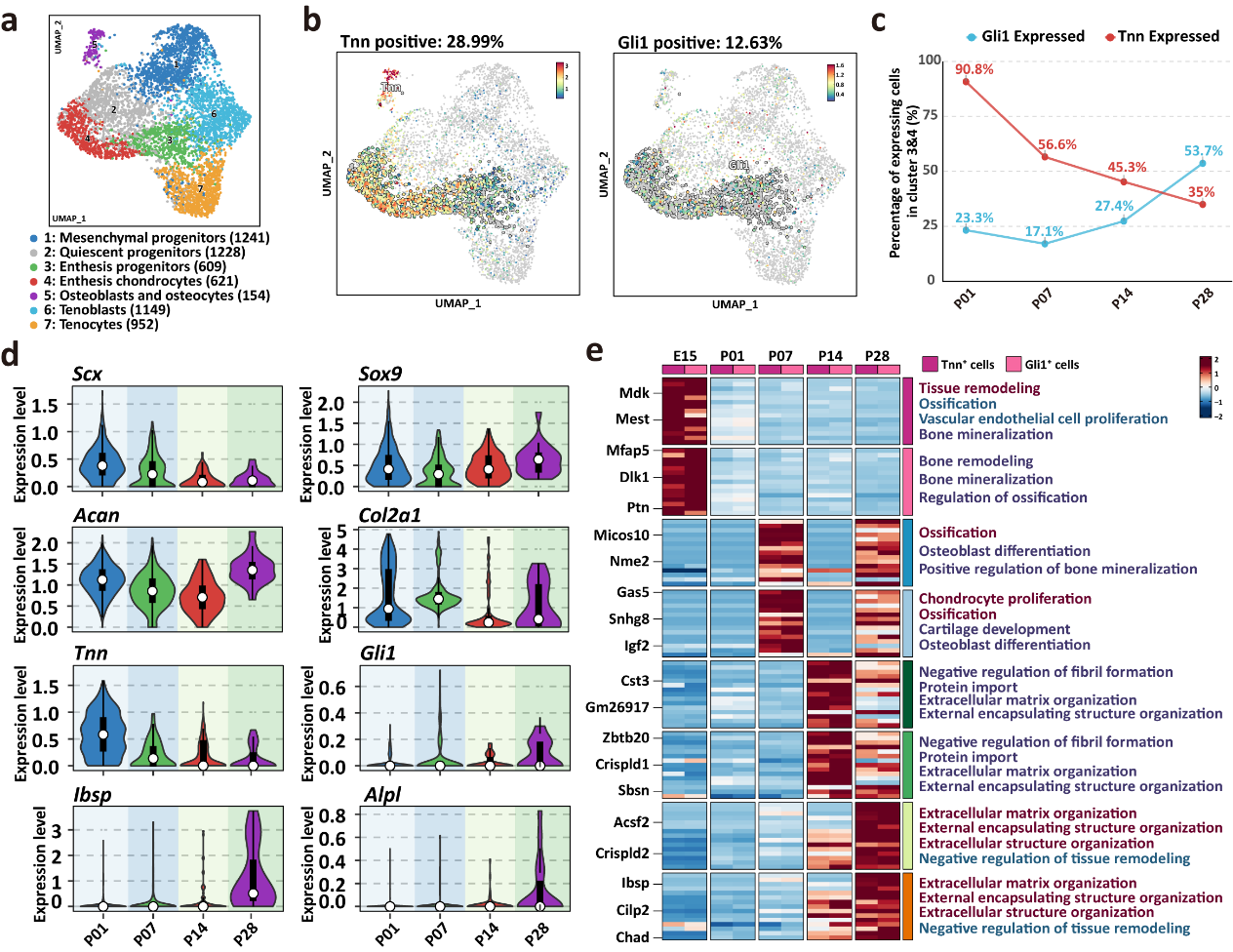
**

**Figure S4. Similarities and differences between Gli1^+^ and Tnn^+^ cells in enthesis chondrogenesis. a** Uniform Manifold Approximation and Projection (UMAP) plot visualizing integrated cell clusters (mesenchymal progenitors, quiescent progenitors, enthesis progenitors, enthesis chondrocytes, osteoblasts and osteocytes, tenoblasts, tenocytes) from normally developing entheses at embryonic day 15 (E15), postnatal day 1 (P1), P7, P14, and P28. **b** Feature plots to show the expression of *Tnn* and *Gli1* across all clusters. **c** Line plots to show the positive rate change of *Tnn* and *Gli1* across all clusters at postnatal day 1 (P1), P7, P14, and P28. **d** Feature plots to show the expressions of genes known as markers for chondrogenesis and biomineralization at different timepoints. **e** Heatmap visualization to show the comparisons of genes and GO terms enriched in *Tnn*^+^ and *Gli1*^+^ enthesis progenitors and enthesis chondrocytes at different timepoints.


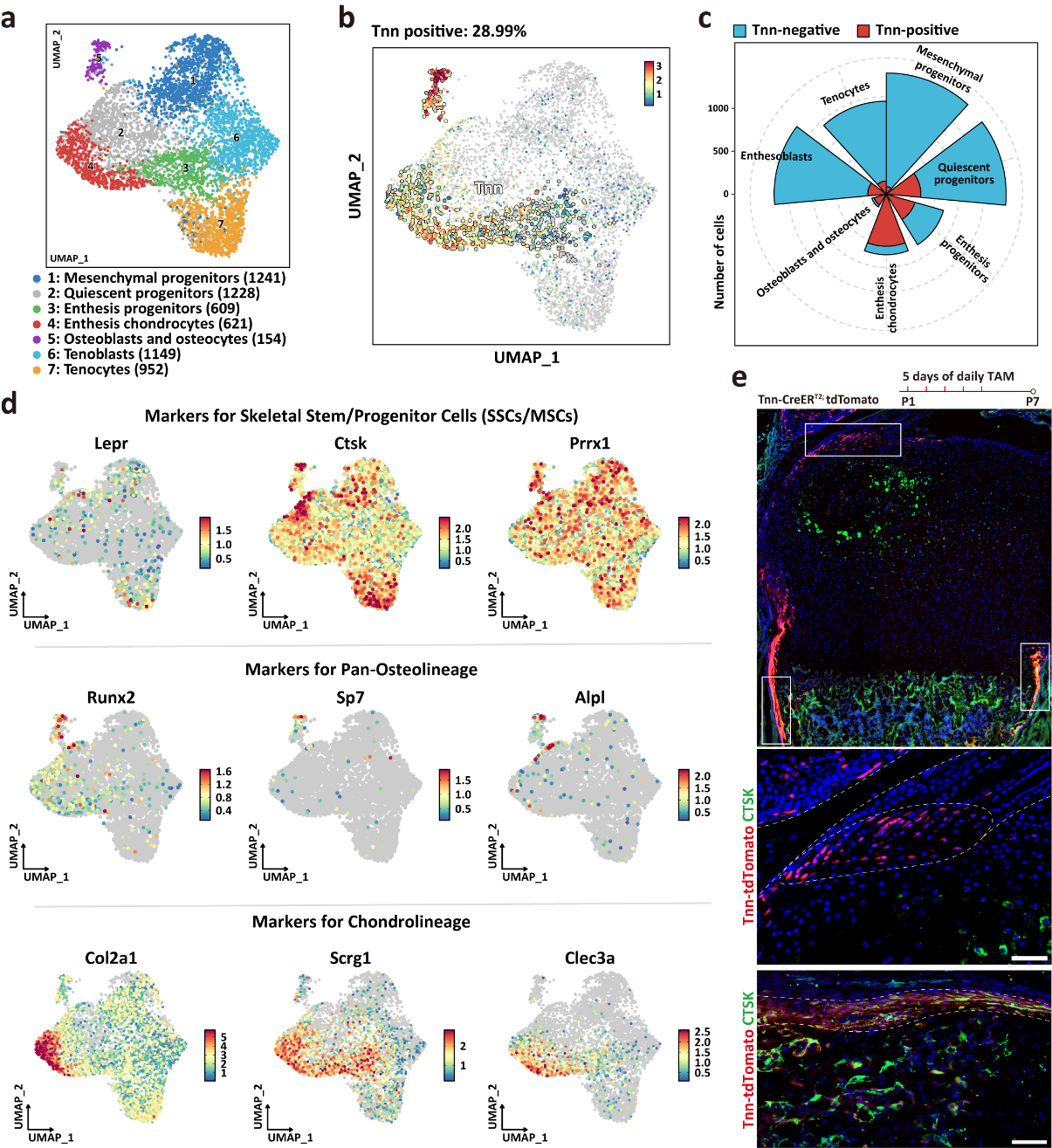


**Figure S5. Two spatially and phenotypically distinct populations of Tnn-tdTomato⁺ cells. a** Uniform Manifold Approximation and Projection (UMAP) plot visualizing integrated cell clusters (mesenchymal progenitors, quiescent chondro-progenitors, enthesis progenitors and chondrocytes, osteoblasts and osteocytes, tenoblasts, tenocytes) from normally developing entheses at embryonic day 15 (E15), postnatal day 1 (P1), P7, P14, and P28. **b, c** Feature plots and bar plots highlighting the expression of Tnn across all clusters and the positive rate of Tnn^+^ cells in each cluster. **d** Feature plots illustrating the genes known as markers for skeletal stem/progenitor Cells (SSCs/MSCs), osteolineage, and chondrolineage across all cell clusters. **e** P7 Tnn-Cre^ERT2^; tdTomato sections co-stained with an antibody for Cathepsin K (CTSK), a marker associated with the periosteal/osteogenic lineage. Scale bars = 50 μm.


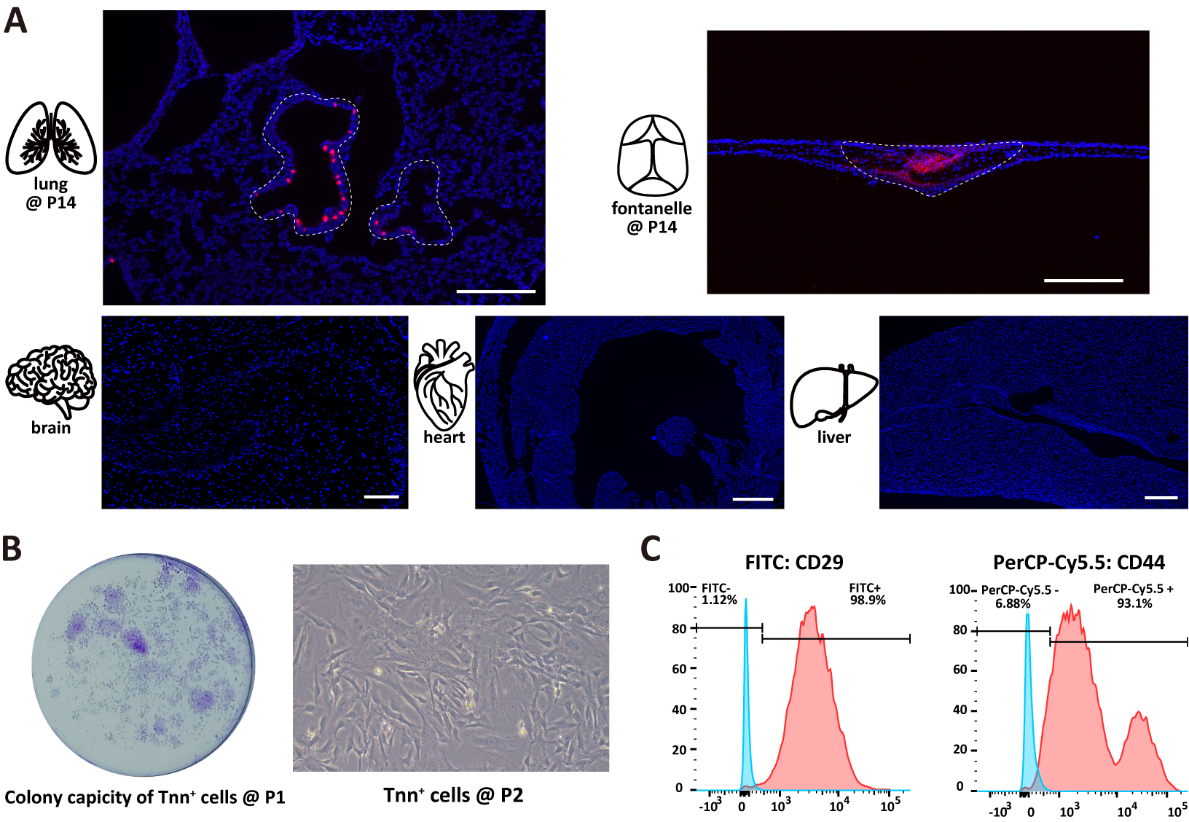


**Figure S6. In vivo distribution of and in vitro potency of Tnn^+^ cells. a** Tnn-Cre^ERT2^; tdTomato mice received 5 daily injections of TAM since P1 until sacrifice on P7 or P14. Tnn^+^ cells were abundantly distributed in the small bronchi of the lung (P14), the fontanelle (P14). While no Tnn^+^ cells were observed in brain, heart, or liver. Scale bars = 100 μm. **b** Tnn^+^ enthesis progenitors exhibited strong clonogenicity. **c** More than 95% of the Tnn^+^ enthesis progenitors isolated from P1 entheses expressed progenitor markers CD29 and CD44.


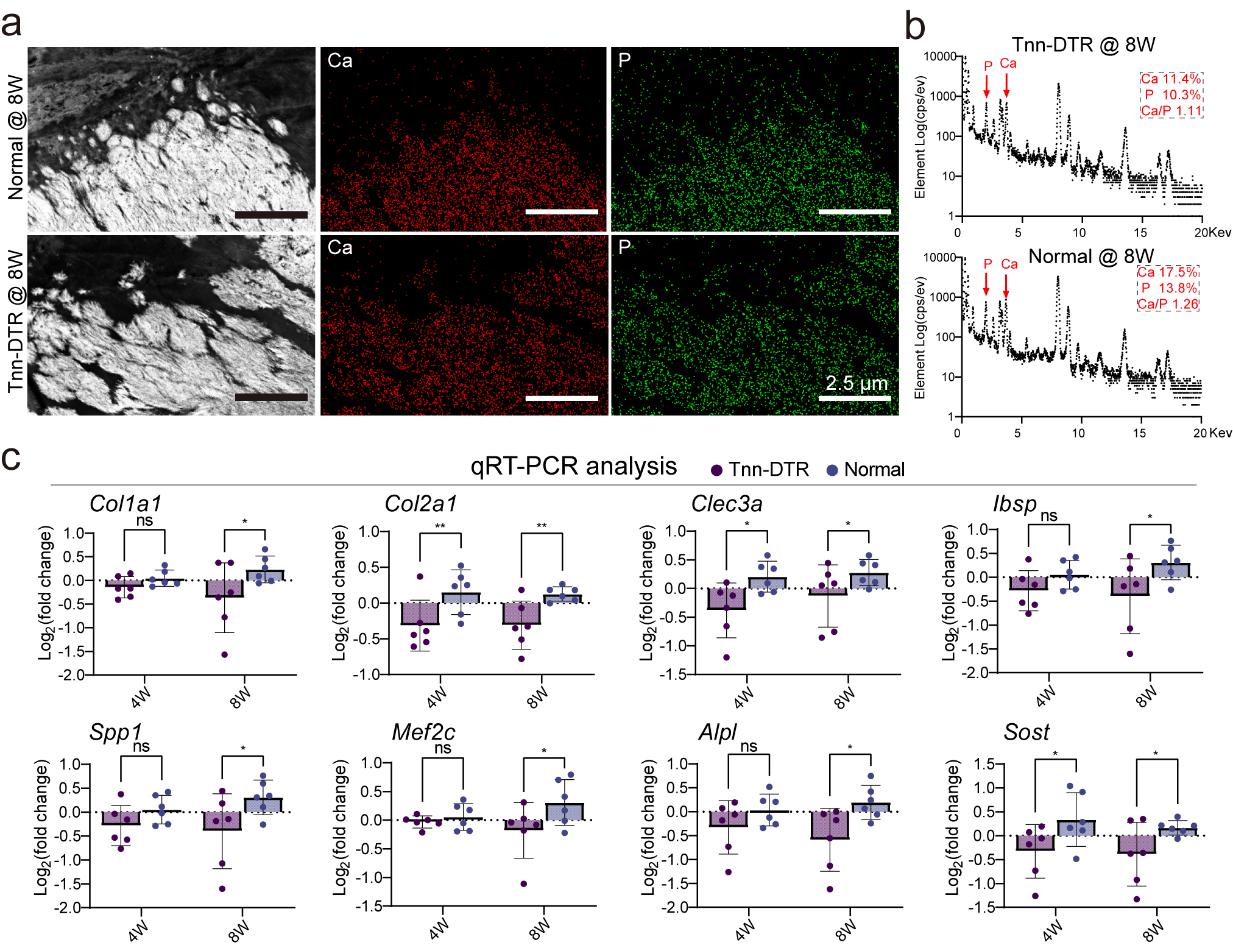


**Figure S7. Ablating the Tnn+ enthesis progenitors inhibits fibrocartilage development. a, b** Quantitative analysis of mineralization-related elements including Calcium (Ca) and Phosphorus (P) in the entheseal fibrocartilage of control and Tnn-DTR groups at 4 and 8 weeks postnatally, measured by transmission electron microscopy (TEM) coupled with energy-dispersive X-ray spectroscopy (EDS). Scale bars = 25 nm. **c** Relative mRNA expression levels, determined by qRT-PCR, of genes involved in collagen synthesis (*Col1a1*, *Col2a1*), chondrocyte hypertrophic differentiation (*Clec3a*, *Mef2c*), and biomineralization (*Ibsp*, *Spp1*, *Alpl*, *Sost*), comparing control and Tnn-DTR groups at specified postnatal time points (n = 6/group). Data are presented as mean ± SEM. Two-way ANOVA and Tukey post-hoc test. Data presented as mean ± SEM. *P < 0.05, **P < 0.01.


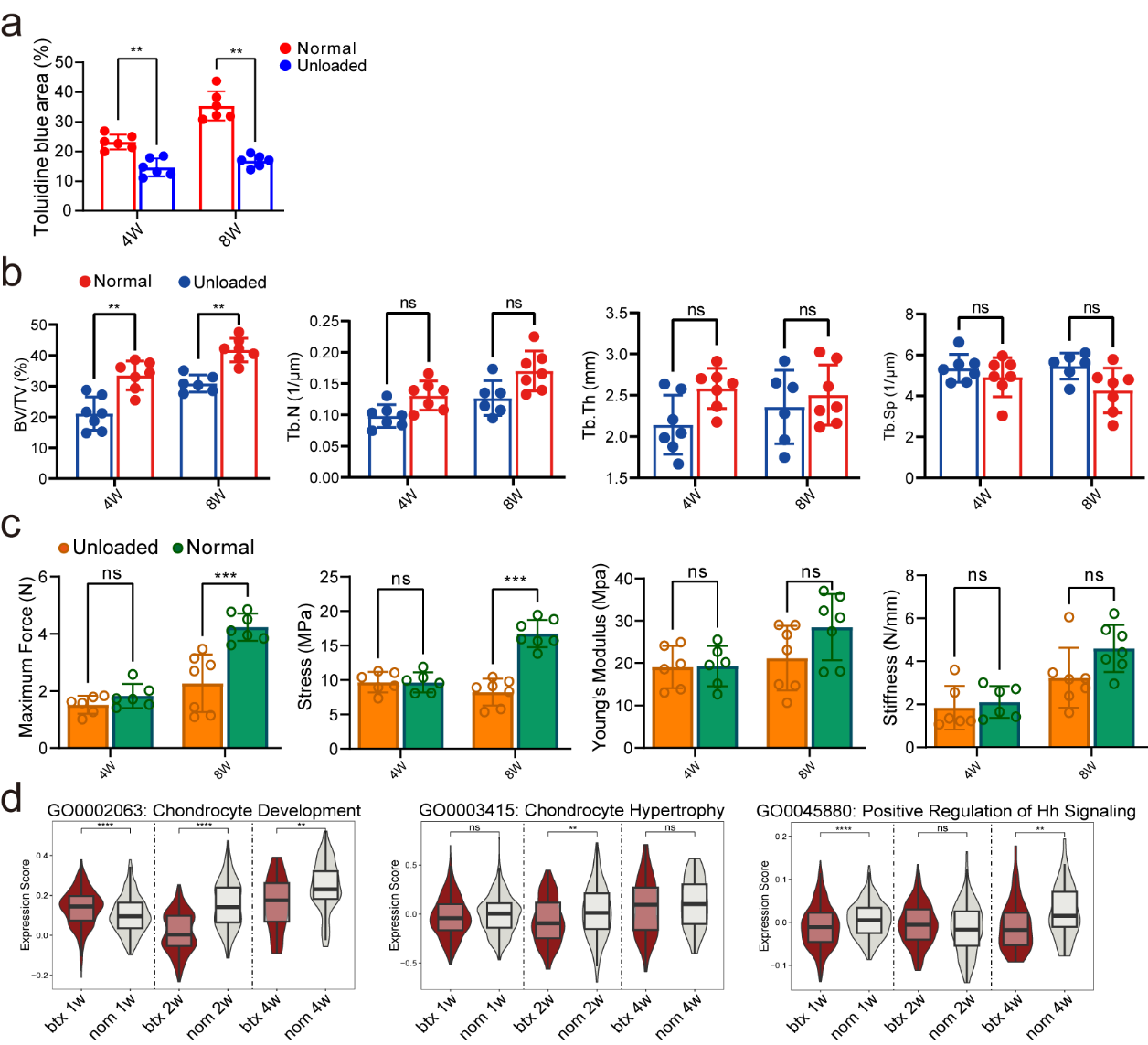


**Figure S8. Enthesis chondrogenesis is hampered after tendon unloading. a** Comparisons of toluidine blue stained area from the normal groups and the unloaded groups (n = 6/group). Mann-Whitney U test. Data presented as mean ± SEM. **P < 0.01. **b** Comparison of bone morphometrics of tendon entheses of mice from the normal groups and the unloaded groups (n = 6-7/group). Mann-Whitney U test. Data presented as mean ± SEM. **P < 0.01. **c** Biomechanical properties of entheses from normal groups and the unloaded groups (n = 6-7/group). Mann-Whitney U test. Data presented as mean ± SEM. ***P < 0.001.


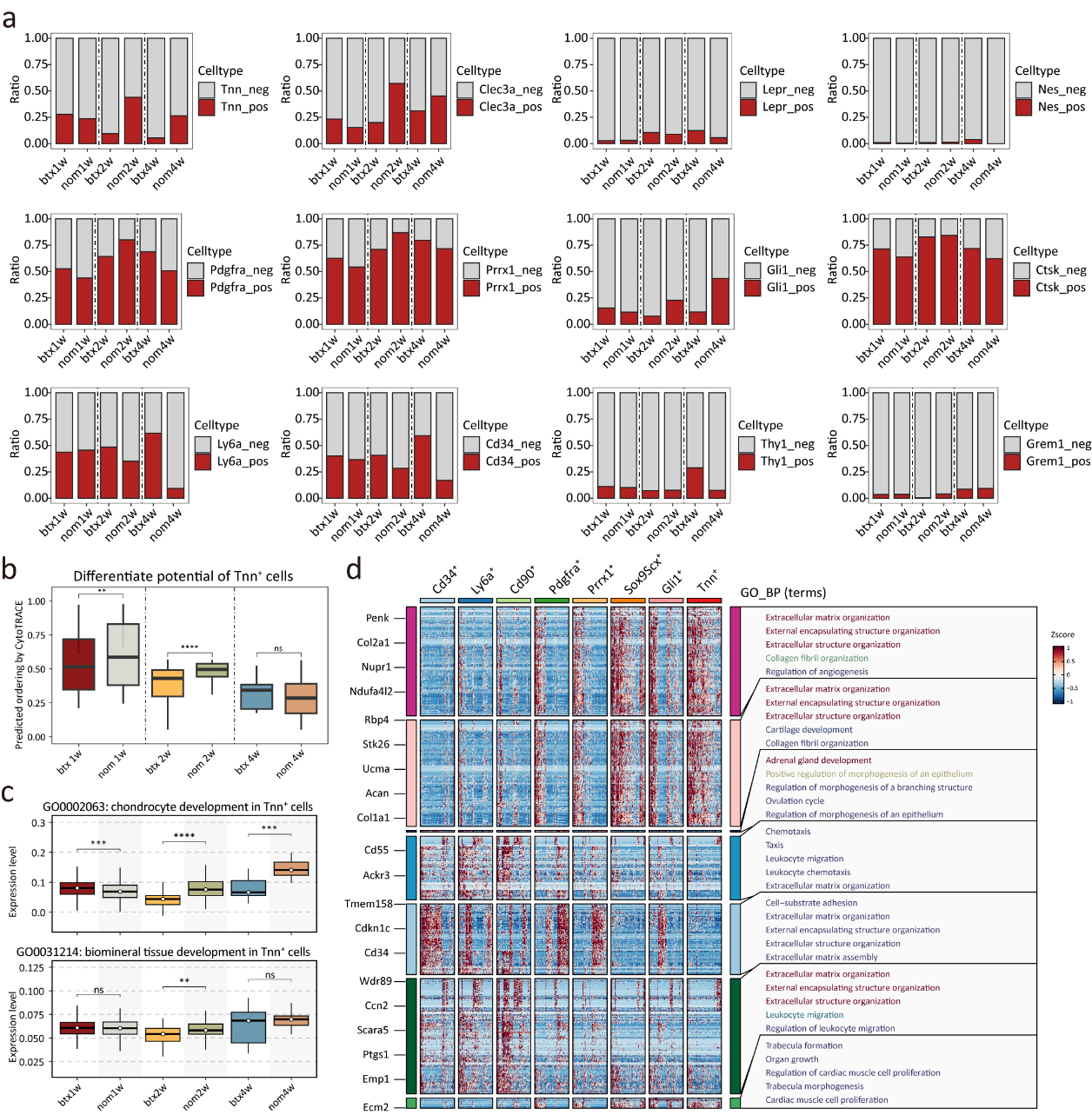


**Figure S9.** **Comparison of the number of Tnn+ cells with other classical stem cell subpopulations in enthesis chondrogenic trajectory. a** Proportion of positive expressed known stem cell markers within enthesis fibrocartilage differentiation trajectory. **b** Comparison of differential potential of *Tnn*^+^ enthesis progenitors between the normal developed groups and the unloaded groups. **c** Comparison of chondrogenic related expressions of *Tnn*^+^ enthesis progenitors between the normal developed groups and the unloaded groups. **d** Heatmap visualization to show the comparisons of genes and GO terms enriched in *Tnn*^+^ enthesis progenitors and other stem cell clusters. * P<0.05, ** P<0.01, *** P<0.001, **** P<0.0001.


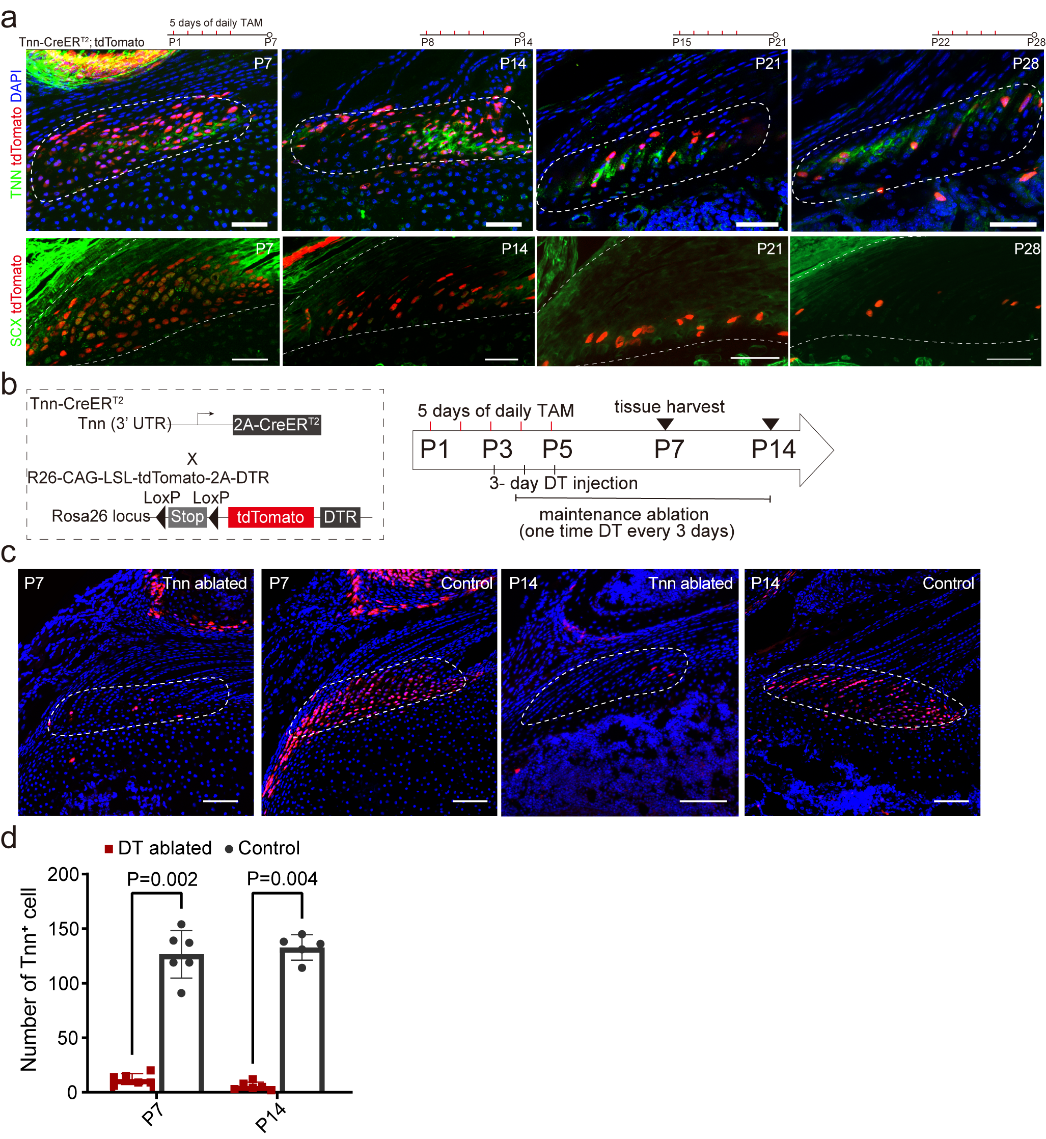


**Figure S10.** **Validation of Tnn reporter with immunohistochemistry and the efficiency of Tnn ablation model. a** Co-stained immunofluorescent images of Tnn-tdTomato (red) with TNN (green) or SCX (green) from Tnn-CreERT2; tdTomato mice. Five consecutive tamoxifen injection was administered from P1-P5, P8-P12, P15-P19, and P22-P26, with tissues harvested for analysis on P7, P14, P21, and P28, respectively. (n = 3 mice per group). Scale bars = 50 μm. **b** Tnn-Cre^ERT2^ mice were mated with R26^CAG-LSL-tdTomato-2A-DTR^ mice. Cre recombination was induced starting at P1 with five consecutive daily intraperitoneal injections of tamoxifen (50 mg/kg body weight). When two days after the first tamoxifen injection (at P3), mice received 3 days of first phase daily injection of Diphtheria Toxin (DT; 5ng/g body weight, Sigma-Aldrich) into the right shoulder, followed by one time DT injection every 3 days until sacrifice at P7 and P14, respectively. Tnn-CreERT2; tdTomato-iDTR mice without DT injection served as controls. **c** Representative lineage tracing images of *Tnn*^+^ cells at entheses from *Tnn*^+^ cell ablated and control mice (n = 5-6 mice per group). Scale bars = 50 μm. **d** Cell number comparisons of *Tnn*^+^ cells from *Tnn*^+^ cell ablated and control mice (n = 5-6 mice per group). Mann-Whitney U test. Data presented as mean ± SEM. P value list in graph.


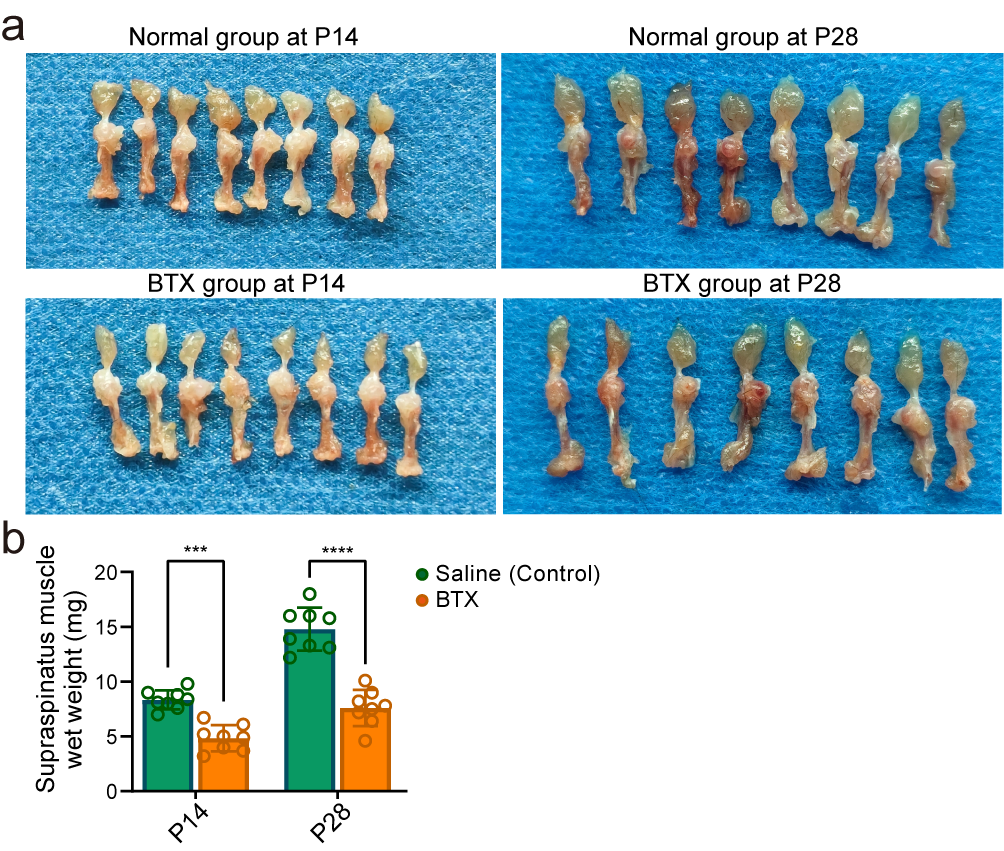


**Figure S11.** **Validation of** **BTX-induced supraspinatus muscle atrophy. a** Representative gross morphology of supraspinatus muscles from shoulders injected with either Botulinum Toxin A (BTX) or saline (control) (n = 8/ group). **b** Quantification of supraspinatus muscle wet weight in the BTX and saline groups (n = 8/ group). All muscle samples were weighed right after harvest and placed in the same room environment. Data are presented as mean ± SEM. Two-way ANOVA and Tukey post-hoc test. Data presented as mean ± SEM. ***P < 0.001, ****P < 0.0001.


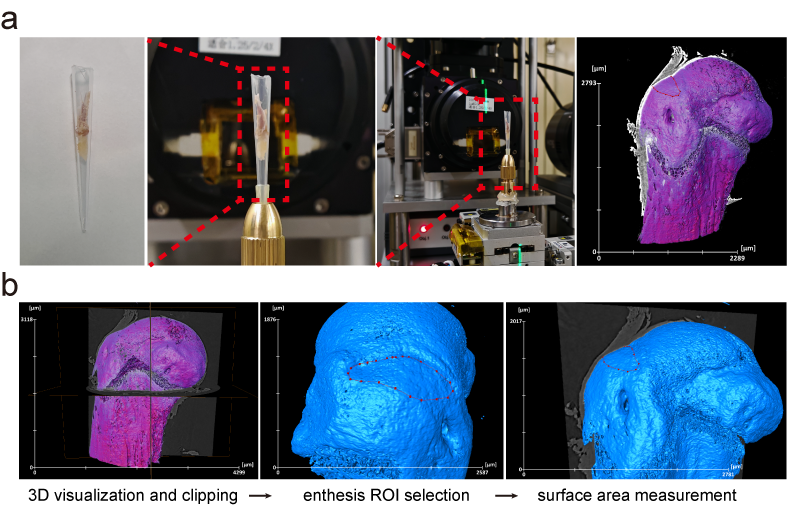


**Figure S12. Schematic diagram of the stabilization setup and enthesis surface definition in SR-μCT experiments. a** The supraspinatus-humerus complex was firmly wedged into a 200 µL pipette tip, with the bony portion of the sample secured within the main body of the tip. Then the pipette tip acted as a custom sample holder and was securely mounted onto the scanner's rotating stage. Fixation to the stage was achieved by using a combination of a set screw and hot-melt adhesive, creating a rigid and stable connection. **b** The cross-sectional area of the enthesis, also defined as the supraspinatus tendon insertion footprint area was outlined in the Amira 3D processing software and converted into a 3D surface and automatically calculated with its surface area.


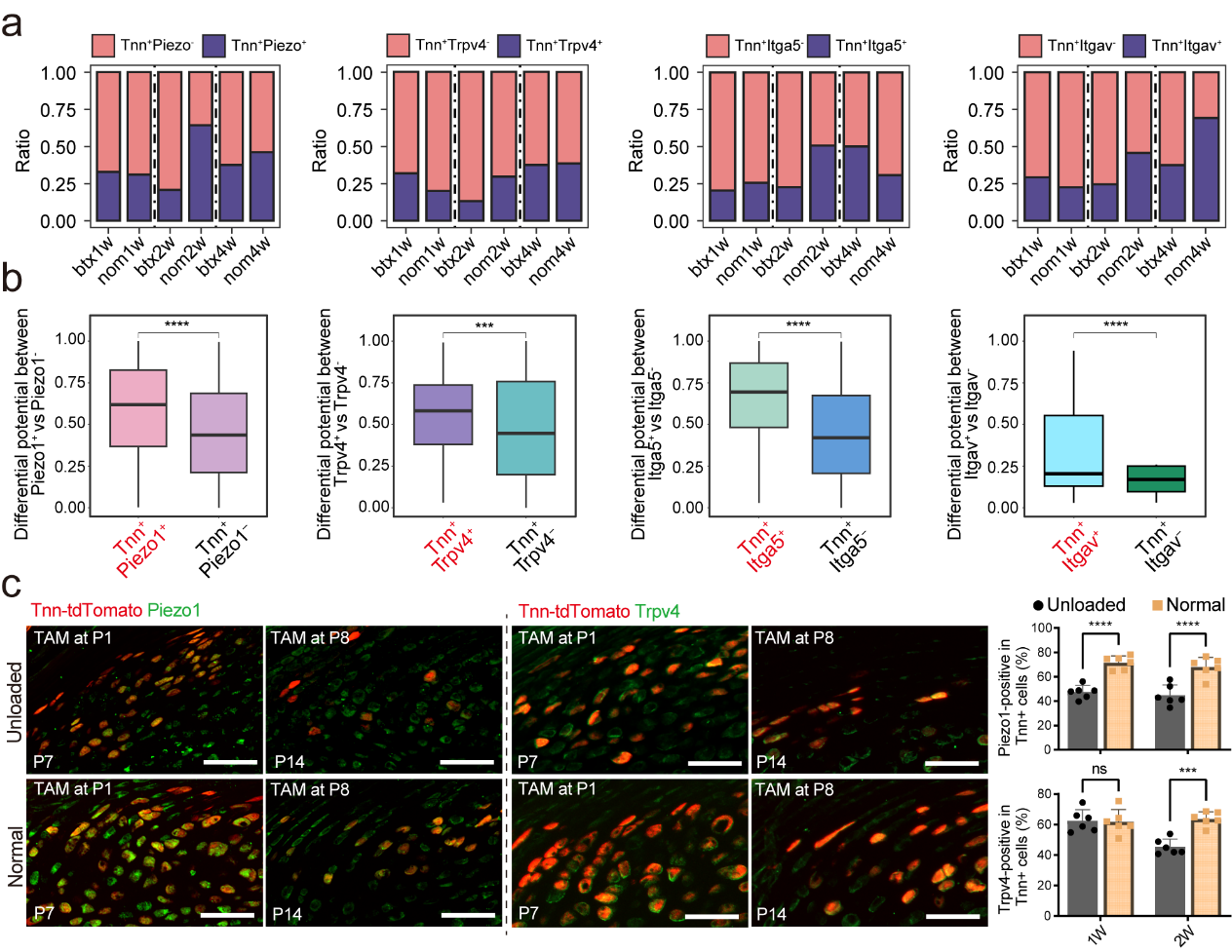


**Figure S13. Expressions of previous reported mechanosensitive channels in Tnn^+^ cells were significantly decreased after the loss of mechanical stimulation. a** Single cell analysis of the proportion of positive expression of mechanosensitive molecules (*Piezo1*, *Trpv4*, *Igta5*, *Itgav*) in *Tnn*^+^ enthesis progenitors and chondrocytes (cluster 3 and 4) from normal developed and the unloaded entheses. **b** Comparison of differentiation capabilities between *Piezo1*, *Trpv4*, *Itga5*, and *Igtav* positive cells in *Tnn*^+^ enthesis progenitors and chondrocytes (cluster 3 and cluster 4 in Fig. 2b). **c** *Tnn*^+^ enthesis progenitors stained with PIEZO1 and TRPV4 antibody (n = 6 mice per group). Five consecutive tamoxifen injection was administered from P1-P5, P8-P12, with tissues harvested for analysis on P7 and P14. Two-way ANOVA and Tukey post-hoc test. Data presented as mean ± SEM. *P < 0.05, **P < 0.01, ***P < 0.001.
